# Trafficking of mitochondrial double-stranded RNA from mitochondria to the cytosol

**DOI:** 10.26508/lsa.202302396

**Published:** 2024-07-02

**Authors:** Matthew R Krieger, Melania Abrahamian, Kevin L He, Sean Atamdede, Hesamedin Hakimjavadi, Milica Momcilovic, Dejerianne Ostrow, Simran DS Maggo, Yik Pui Tsang, Xiaowu Gai, Guillaume F Chanfreau, David B Shackelford, Michael A Teitell, Carla M Koehler

**Affiliations:** 1 Department of Chemistry and Biochemistry, UCLA, Los Angeles, CA, USA; 2 Department of Pathology, Children’s Hospital Los Angeles, Los Angeles, CA, USA; 3 Pulmonary and Critical Care Medicine, David Geffen School of Medicine, UCLA, Los Angeles, CA, USA; 4 Jonsson Comprehensive Cancer Center, UCLA, Los Angeles, CA, USA; 5 Department of Pathology, Keck School of Medicine, University of Southern California, Los Angeles, CA, USA; 6 Molecular Biology Institute, UCLA, Los Angeles, CA, USA; 7 Department of Pathology and Laboratory Medicine, UCLA, Los Angeles, CA, USA; 8 Broad Stem Cell Research Center, UCLA, Los Angeles, CA, USA; 9 NanoSystems Institute, UCLA, Los Angeles, CA, USA

## Abstract

In addition to mitochondrial DNA, mitochondrial double-stranded RNA (mtdsRNA) is exported from mitochondria. However, specific channels for RNA transport have not been demonstrated. Here, we begin to characterize channel candidates for mtdsRNA export from the mitochondrial matrix to the cytosol. Down-regulation of SUV3 resulted in the accumulation of mtdsRNAs in the matrix, whereas down-regulation of PNPase resulted in the export of mtdsRNAs to the cytosol. Targeting experiments show that PNPase functions in both the intermembrane space and matrix. Strand-specific sequencing of the double-stranded RNA confirms the mitochondrial origin. Inhibiting or down-regulating outer membrane proteins VDAC1/2 and BAK/BAX or inner membrane proteins PHB1/2 strongly attenuated the export of mtdsRNAs to the cytosol. The cytosolic mtdsRNAs subsequently localized to large granules containing the stress protein TIA-1 and activated the type 1 interferon stress response pathway. Abundant mtdsRNAs were detected in a subset of non–small-cell lung cancer cell lines that were glycolytic, indicating relevance in cancer biology. Thus, we propose that mtdsRNA is a new damage-associated molecular pattern that is exported from mitochondria in a regulated manner.

## Abstract (precis)

Mitochondrial double-stranded RNA is a new signaling molecule that, likely under stress conditions, is exported to induce a type 1 interferon response, thus mimicking a viral response. This export pathway is detected in a subset of lung cancer cell lines.

## Introduction

Whereas the vast majority of mitochondrial proteins are encoded within the nucleus and imported into mitochondria by mechanisms studied in great detail (Chacinska et al, 2009; Becker et al, 2019), less is known about the transport of nucleic acids across the mitochondrial membranes. Nucleic acids that are transported include mitochondrial DNA (mtDNA), nuclear-encoded tRNAs and non-coding RNAs, mitochondrial double-stranded RNAs (mtdsRNA), and viral RNAs. Thus, a large cohort of nucleic acids cross the mitochondrial outer and inner membranes. Cytosolic mtDNA and recently identified mtdsRNA function as damage-associated molecular patterns (DAMPs) to signal mitochondrial stress (Dhir et al, 2018; Wu et al, 2021) to the rest of the cell.

mtDNA is released from mitochondria as an endogenous trigger for inflammation, activating pro-inflammatory and type 1 IFN (IFN-1) responses (West & Shadel, 2017). The specific manner of release is not known, but potential mechanisms include mitochondrial fragmentation (West et al, 2015), mitophagy (Zhong et al, 2016), apoptosis with mitochondrial herniation (Rongvaux et al, 2014; White et al, 2014; McArthur et al, 2018), and the membrane permeability transition pore (Garcia & Chavez, 2007; Xian et al, 2022), among others. To date, a specific channel for the direct export of mtDNA has not been demonstrated experimentally (West & Shadel, 2017), but recent studies suggest that VDAC and BAK/BAX oligomers with the mitochondrial permeability transition pore may facilitate mtDNA export (Kim et al, 2019; Yu et al, 2020; Xian et al, 2022).

Viral RNAs also translocate into mitochondria, but the specific pathways are not known. Human cytomegalovirus has a 2.7-kilobase RNA, the β2.7 transcript, that is imported into mitochondria and interacts with complex I to regulate apoptosis (Reeves et al, 2007). The HIV-1 RNA transcripts reside in mitochondria, potentially to compromise mitochondrial function (Somasundaran et al, 1994). Interestingly, computational methods for RNA localization predict that the SARS-CoV-2 RNA genome and subgenomic transcripts (sgRNA) are enriched in the mitochondrial matrix and the nucleolus (Wu et al, 2020). In contrast, these computational methods indicated that transcripts from other SARS viruses did not reside in the mitochondrial matrix. Although these computational predictions have not been verified, support for viral RNA import into mitochondria is warranted as the mitochondria are a critical target in viral replication and propagation strategies.

A variety of cellular RNAs are imported into mitochondria (Sieber et al, 2011; Wang et al, 2012a; Jeandard et al, 2019). Every organism imports at least one nuclear-encoded tRNA, in some cases, that is only required during stress (Simpson & Shaw, 1989; Hancock & Hajduk, 1990; Rubio et al, 2008; Kamenski et al, 2010). Structural RNAs that are imported include the *5S* rRNA, *H1* RNA of RNase P, and RNase *MRP* RNA component that are potentially involved in RNA processing and translation in the mitochondrion (Mercer et al, 2011; Smirnov et al, 2011; Antonicka et al, 2013; Jourdain et al, 2013). Numerous long non-coding RNAs and microRNAs have been localized to mitochondria, and potential functions include storage and regulation of transcription and translation (Dong et al, 2017). Given the large number of microRNAs, the microRNA subset that resides in mitochondria is referred to as miRNAs of nuclear or mitochondrial origin that are localized in mitochondria (MitomiRs) and have a wide variety of proposed functions in mitochondrial gene expression and regulation (Bandiera et al, 2013; Geiger & Dalgaard, 2017).

In addition to import, initial results in our collaborative study with the Proudfoot group have shown that loss of PNPase resulted in the export of mtdsRNA to the cytosol (Dhir et al, 2018). The mtdsRNAs are novel DAMPs that activate MDA5 and RIG-I and trigger the antiviral IFN-1 response pathway (Dhir et al, 2018). Exported mtdsRNAs have also been characterized in a fly model that lacks functional mitochondrial poly(A) polymerase (Pajak et al, 2019).

Although the transport mechanism of RNAs across mitochondrial membranes is not well understood, numerous studies indicate different RNA species use different translocation pathways, suggesting that RNA import pathways may have been developed independently several times during evolution (Salinas et al, 2008). A common feature for RNA import is the requirement of a membrane potential and an energy form such as ATP. The necessity of energy is in the form of ATP hydrolysis and maintenance of the mitochondrial inner membrane potential. Additional properties of RNA import mechanisms may vary based on the RNA species and the organism. Tarassov and colleagues have shown that tRNA^Lys^ import in yeast requires enolase and cytosolic precursors of aminoacyl-tRNA synthetases (Tarassov et al, 1995; Kamenski et al, 2010). Mitochondrial PNPase has been shown to act as a gatekeeper to mediate the import of a subset of RNAs into mitochondria (Wang et al, 2010; Wang et al, 2012b; Vedrenne et al, 2012).

The export mechanism of mtdsRNAs from mitochondria highlights a new pathway in which mtdsRNAs are exported from mitochondria that maintain a membrane potential (Chen et al, 2006). A central question is how the mitochondria remain functional while the large mtdsRNA cargos are exported. Here, we build on our earlier collaboration (Dhir et al, 2018) and begin to outline a pathway for the export of mtdsRNAs. At the outer membrane, VDAC and BAK/BAX, which have been implicated in mtDNA release (McArthur et al, 2018; Tigano et al, 2021), are key components, whereas the inner membrane components prohibitin 1 and 2 (PHB1/PHB2) are candidates that mediate release across the inner membrane.

## Results

### PNPase but not SUV3 is required for the export of mtdsRNAs to the cytosol

In a previous collaborative study, BAK and BAX participated in mtdsRNA export (Dhir et al, 2018), akin to the release of mtDNA during apoptosis (Rongvaux et al, 2014; White et al, 2014; McArthur et al, 2018). However, as BAK and BAX are typically associated with apoptosis and patients with PNPase mutations survive until late in life (Vedrenne et al, 2012; von Ameln et al, 2012; Eaton et al, 2018; Sato et al, 2018; Rius et al, 2019), activation of a classic apoptotic pathway is unlikely. We sought to characterize the mtdsRNA export pathway in detail. We used immunofluorescence microscopy with a monoclonal antibody (J2) that is specific for dsRNA and is widely used to detect viral dsRNA in plant and animal cells (Weber et al, 2006). The monoclonal J2 antibody was developed against the dsRNA killer virus of *Saccharomyces cerevisiae* and recognizes dsRNAs that are ∼40–50 nucleotides or longer (Schonborn et al, 1991).

**Figure S1.**
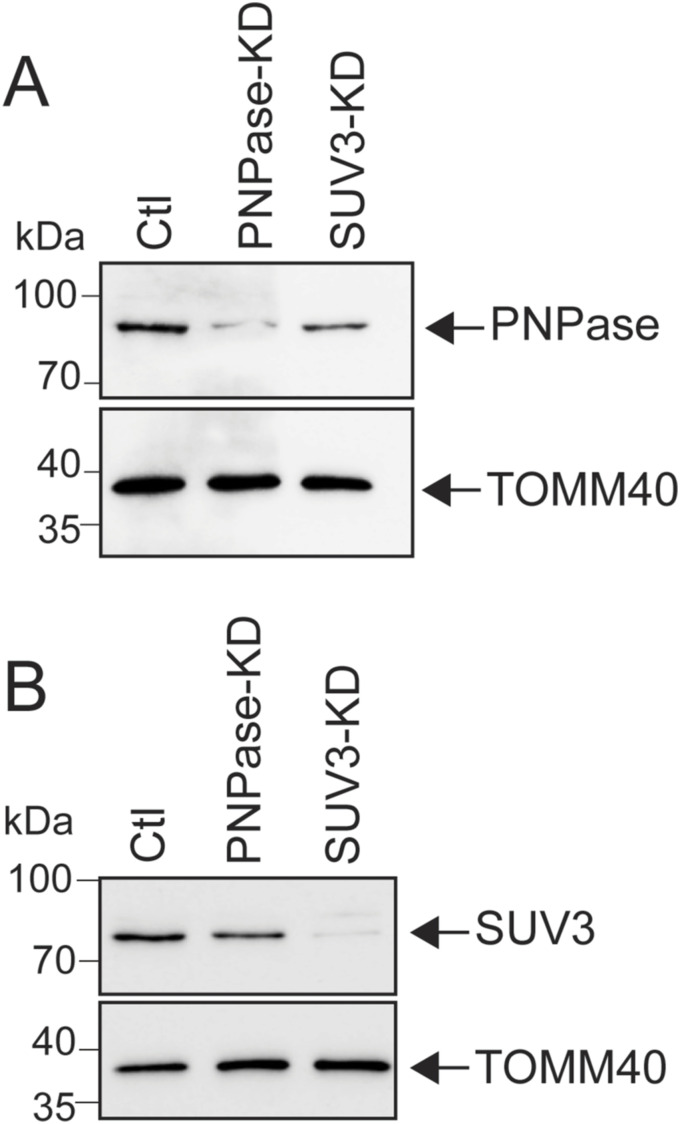
Control experiments to validate RNAi of SUV3 and PNPase. **(A)** Cells were treated with RNAi constructs to knock down PNPase or SUV3. A negative control (Ctl) that consisted of a non-targeting RNAi construct was included. After 72 h, PNPase KD was confirmed by immunoblot analysis. TOMM40 was included as a loading control. **(A, B)** As in “(A),” SUV3 levels were assessed by immunoblotting.

We first confirmed a linear pathway in which SUV3 knockdown (KD) followed by PNPase KD was required for release of mtdsRNAs to the cytosol (Dhir et al, 2018). An RNAi KD strategy was used instead of genomic deletion for PNPase (Fig S1A) because cells that lack PNPase lose mtDNA and become rho null (ρ^0^) (Shimada et al, 2018). SUV3 was also knocked down with RNAi (Fig S1B). Previous studies have suggested that PNPase and SUV3 form a complex (Borowski et al, 2013), suggesting that their expression may be co-dependent However, we show that PNPase and SUV3 protein levels are not dependent upon each other (Fig S1A and B), indicating that these proteins are not bona fide partners. As a negative control (Ctl), a non-targeting RNAi construct did not affect the expression of PNPase or SUV3.

**Figure 1.**
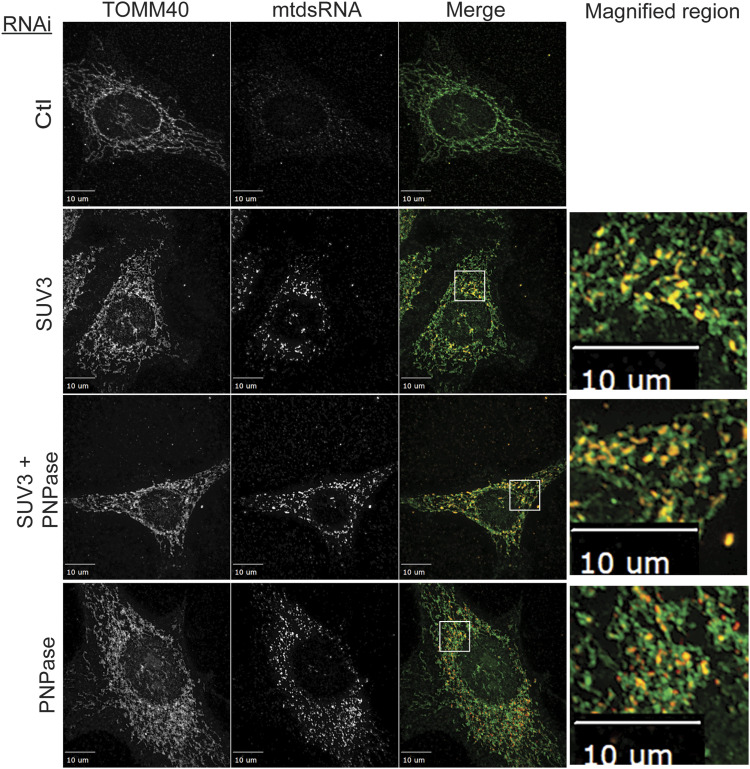
mtdsRNAs accumulate in mitochondria with SUV3 KD, whereas mtdsRNAs localize to the cytosol with PNPase KD. SUV3 (PCC = 0.73), PNPase (PCC = 0.55), and the combination (PCC = 0.69) were knocked down (KD) in HeLa cells with the respective RNAi construct followed by fixing on coverslips and imaging after 72 h. The mtdsRNA (red) was investigated by immunofluorescence with the monoclonal J2 antibody. TOMM40 was detected with a polyclonal antibody to mark mitochondria (green), and the images were merged. The right panel contains a magnified portion from the merged image (marked with a white box). As a control (Ctl), the cells were treated with a non-targeting RNAi construct. The bar indicates 10 μm (*n* = 8).

We investigated the subcellular localization of mtdsRNAs when PNPase and/or SUV3 were knocked down in HeLa cells. With the non-targeting RNAi construct (Ctl), mtdsRNAs (marked by red fluorescence) were essentially not detected in mitochondria, which were marked with TOMM40 (green fluorescence) (Fig 1). SUV3 KD resulted in accumulation of mtdsRNAs in mitochondria in small puncta that overlapped with the mitochondrial marker TOMM40. However, a fraction of mtdsRNAs was detected in puncta in the cytosol when PNPase was knocked down (Fig 1). When both PNPase and SUV3 were knocked down, mtdsRNAs mostly remained in mitochondria, indicating that SUV3 is required before PNPase for mtdsRNA release. Pearson’s correlation coefficient (PCC) in ImageJ software was used to quantify co-localization of mitochondria and mtdsRNA (Dunn et al, 2011). PCC values of 0.73, 0.69, and 0.55 were calculated for KD of SUV3, SUV3/PNPase, and PNPase, respectively. Portions of the images were also magnified to show localization of mtdsRNA with respect to mitochondria. Thus, the mtdsRNA seems to specifically show decreased co-localization with mitochondria when PNPase is knocked down.

**Figure S2.**
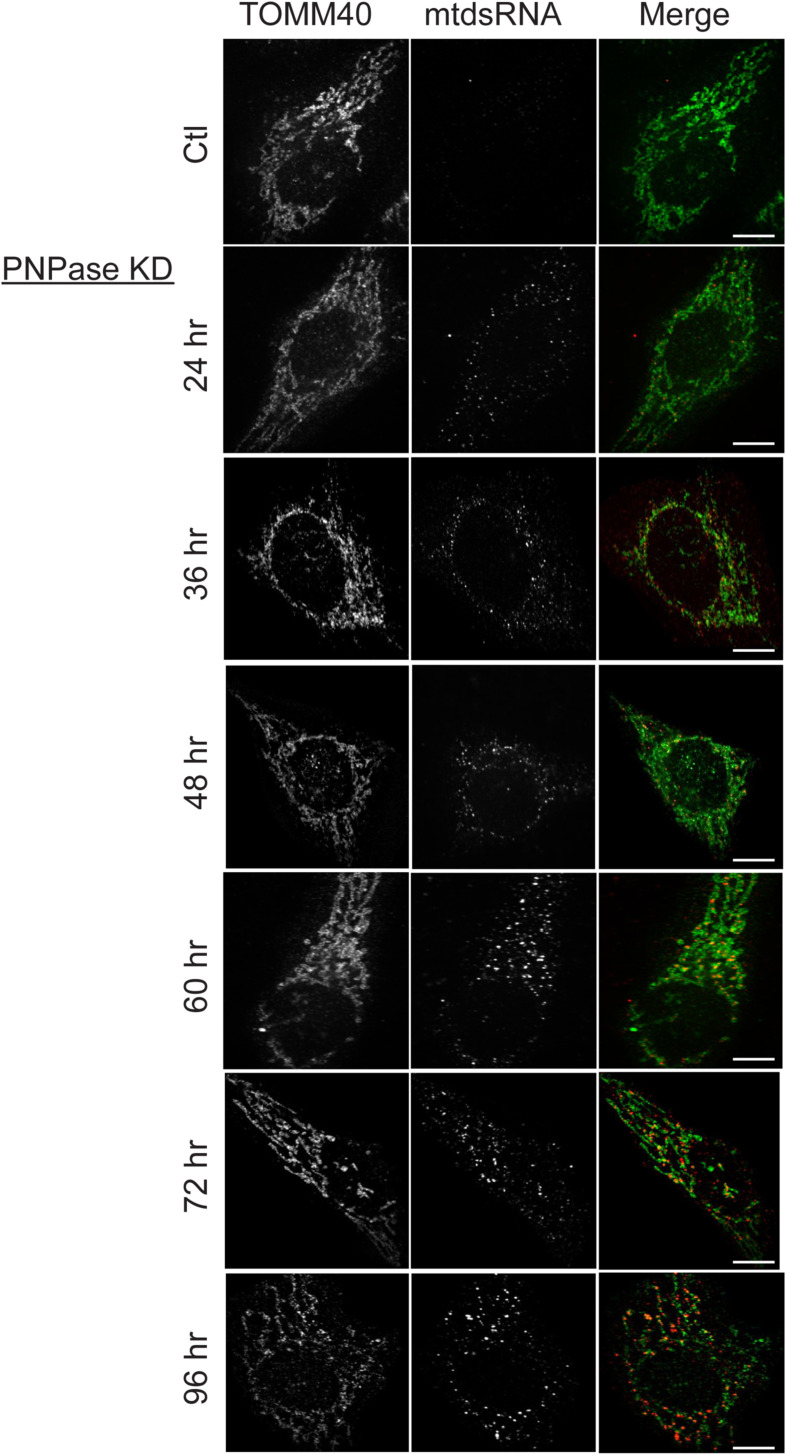
Time-course study of mtdsRNA accumulation and localization in PNPase KD cells. PNPase was knocked down by RNAi as in Fig 1, and cells were fixed at the indicated time points post-transfection and then imaged for mitochondria (TOMM40, green) and mtdsRNA (red). As a control (Ctl), a non-targeting RNAi construct was included. PCC values for 48 h (0.59), 72 h (0.56), and 96 h (0.41) were calculated. The bar indicates 10 μm (*n* = 3).

To determine the time frame in which mtdsRNAs were released, a time-course study was done when PNPase levels were reduced by RNAi (Fig S2). Over 96 h, cells remained healthy. After ∼48 h, mtdsRNAs (marked by red fluorescence) began to move out of mitochondria (marked with green fluorescence) and localize to the cytosol (PCC = 0.59), and at ∼60 h, the mtdsRNAs were found in cytosolic puncta. Based on this time course, 60–72 h post-RNAi treatment (PCC = 0.56) was selected for studies to evaluate mtdsRNA localization. At 96 h, mtdsRNAs were in red puncta and the mitochondrial network appeared largely fragmented (PCC = 0.41). Because we have previously shown that cells without PNPase lose mtDNA, cells will not continue to grow unless adapted to glycolytic media supplemented with uridine (Shimada et al, 2018). In summary, mtdsRNAs were released to the cytosol in a controlled manner.

### The mtdsRNAs partially co-localize with stress granule protein TIA-1, and the type 1 IFN stress response is induced

**Figure S3.**
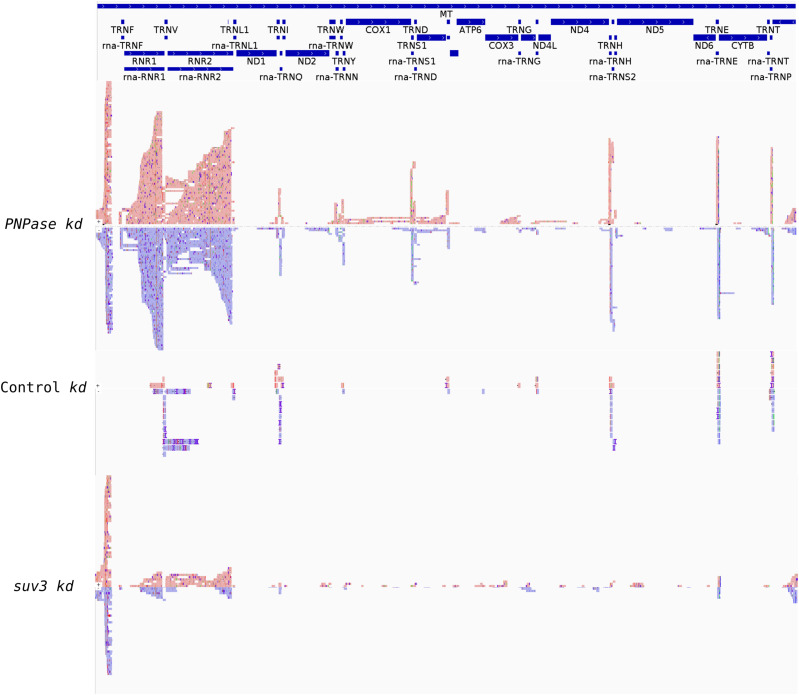
mtdsRNA-sequencing reads are particularly abundant for the ribosomal RNAs and a portion of the D-loop when PNPase is knocked down. The dsRNA was immunoprecipitated with the J2 antibody in cells that were treated with RNAi for PNPase, SUV3, or the control, and then, strand-specific sequencing was performed with the Oxford Nanopore system. The H-strand is shown in red, the L-strand is shown in blue, and they are aligned to the mitochondrial genome.

To independently demonstrate that the dsRNAs detected above originated from mitochondria, we performed immunoprecipitation with the J2 antibody from a total cell lysate followed by Nanopore long-read sequencing. Briefly, the immunoprecipitated RNAs were polyadenylated in vitro and then sequenced using the Oxford Nanopore platform (Liu et al, 2022). After alignment with the mitochondrial genome, the RNA-sequencing profile for cells with PNPase KD showed abundant reads that mapped to both the heavy (H)- and light (L)-strands of the mitochondrial genome, implying the presence of intermolecular dsRNA (Fig S3). Most abundant mtdsRNAs aligned to the ribosomal (r)RNAs and displacement (D)-loop regions. In addition, reads were also detected that aligned with the coding regions of the mitochondrial genome, particularly Cox1 and Cox2. For SUV3 KD, mtdsRNAs were also detected for the rRNAs and D-loop, whereas mtdsRNAs were barely detected for the control cells. Because the L-strand for the rRNAs was abundant in PNPase KD, a transcript for the entire L-strand was generated from the origin of L-strand transcription.

**Figure S4.**
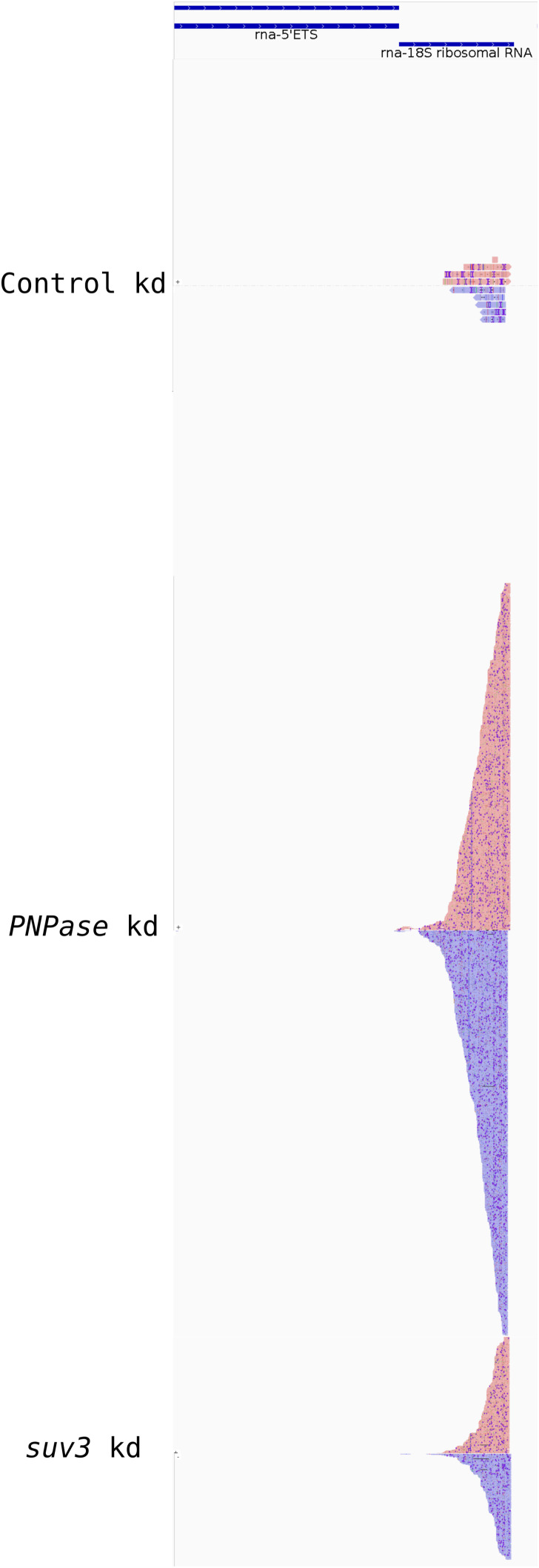
Subset of the dsRNA-sequencing reads aligned with the 18S ribosomal RNA. As in Fig S3, the dsRNA reads were also aligned to the nuclear genome. The only region in which dsRNA was detected was for the 18S ribosomal RNA. The coding strand is shown with red bars and the non-coding strand with the blue bars.

To confirm the specificity of the dsRNAs, we also queried the sequencing reads with the nuclear genome. We only detected dsRNAs that aligned with the coding and non-coding strands for the 18S rRNA when PNPase or SUV3 was knocked down (Fig S4). Previously, dsRNAs for the 18S rRNA have not been reported, but a class of antisense ribosomal siRNAs (risiRNAs) has been identified that functions in ribosomal biogenesis (Zhu et al, 2018). The dsRNA for the 18S rRNA may have a novel role in attenuating cytosolic translation during the IFN-1 response (Wan et al, 2021). In sum, the sequencing analysis confirms that the dsRNAs detected by immunofluorescence were of mitochondrial origin.

**Figure 2.**
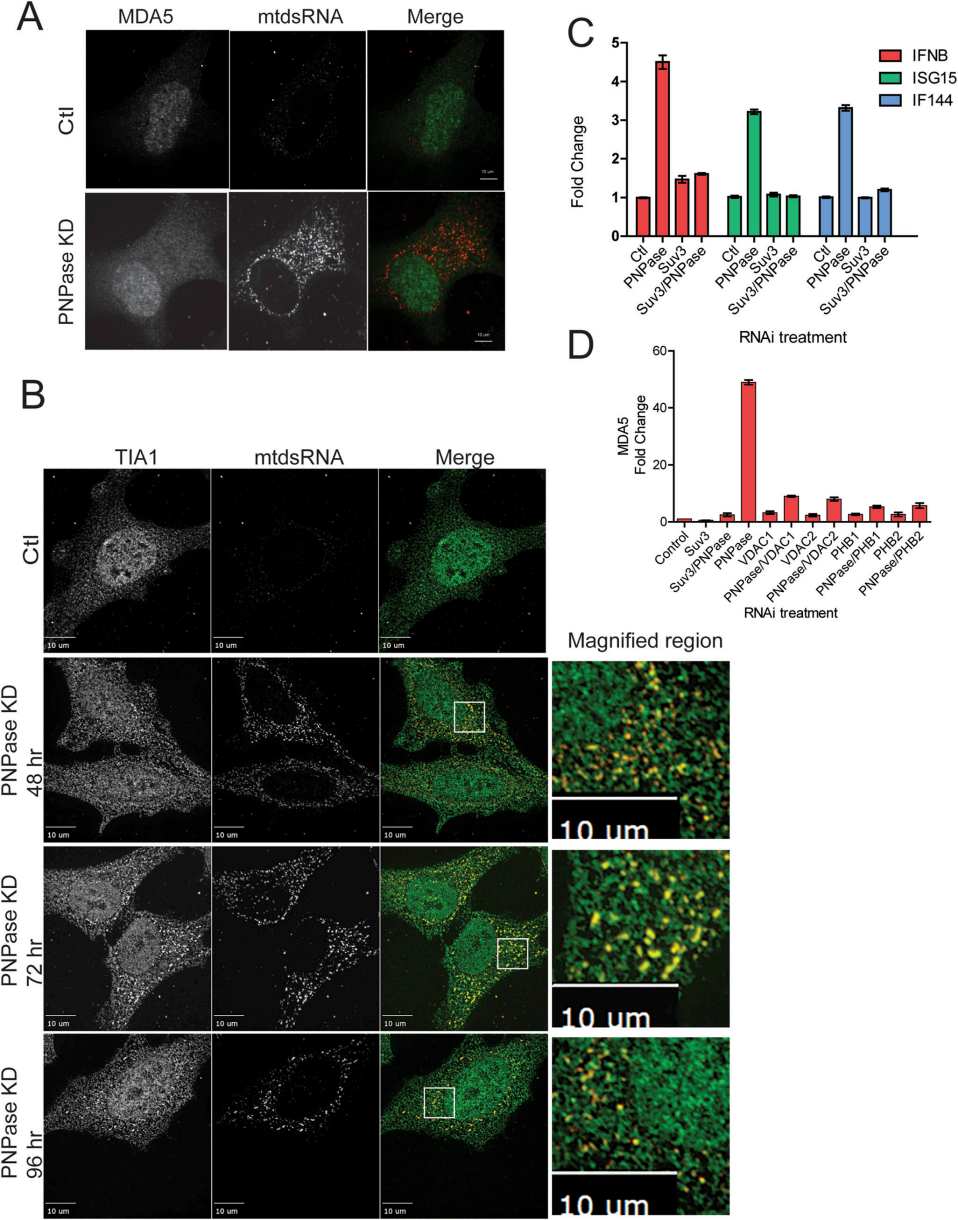
Cytosolic mtdsRNA co-localizes with TIA-1 but not MDA5, and the type 1 IFN pathway is induced. **(A)** PNPase was knocked down by RNAi in HeLa cells, and cells were fixed for immunofluorescence studies at 72 h. MDA5 (green) and mtdsRNA (red) were localized, and the individual images were merged. As a control (Ctl), the cells were treated with a non-targeted RNAi construct. The bar indicates 10 μm (*n* = 3). **(A, B)** As in “(A)” except that TIA-1 (green) and mtdsRNA (red) at 48 (PCC = 0.58), 72 (PCC = 0.66), and 96 (PCC = 0.40) hrs were localized. As a control (Ctl), the cells were treated with a non-targeted RNAi construct. The right panel contains a magnified region from the merged image (marked with a white box). The bar indicates 10 μm (*n* = 3). **(C)** As in Fig 1, SUV3 and PNPase RNAi constructs were applied to HeLa cells, and 72 h post-transfection, qRT-PCR analysis of *IFNB1* and *ISG15* and *IFI44* was performed. Three technical and biological replicates were analyzed. **(C, D)** As in “(C)”, qRT-PCR analysis of *IFIH1* (MDA5 protein) transcript levels was performed 72 h after the indicated RNAi treatments. Three technical and biological replicates were analyzed.

MDA5 and RIG-I are dsRNA sensors in the cytosol that recognize long (>2.0 kb) and short (<2.0 kb) dsRNAs, respectively, and relay the IFN-1 response (Kato et al, 2008; Peisley et al, 2011). Our previous study indicated that knockdown of MDA5 in PNPase-depleted cells mainly blocked the IFN-1 response, but RIG-I also did to a lesser extent (Dhir et al, 2018). We determined whether MDA5 co-localized with mtdsRNA in cells that were depleted for PNPase using immunofluorescence microscopy (Fig 2A). In control cells, mtdsRNA was not detected and MDA5 (green) levels were low. However, PNPase KD resulted in increased MDA5 expression throughout the cell, but it failed to co-localize with mtdsRNA (red). It may not be surprising that MDA5 did not markedly co-localize with cytosolic mtdsRNAs, because published studies have noted that MDA5 did not bind to viral RNAs and the viral RNAs seemed to localize to stress granules (Langereis et al, 2013). Thus, MDA5 likely binds to mtdsRNAs transiently when initiating the IFN-1 response.

Given that mtdsRNAs are present in large structures that are reminiscent of cytosolic stress granules, we tested whether mtdsRNAs co-localized with the RNA binding protein TIA-1, which promotes the assembly of stress granules (Gilks et al, 2004; Asadi et al, 2021). A subset of TIA-1 (green) seemed to co-localize with mtdsRNAs (red) in large puncta at 72 h post-PNPase KD (Fig 2B), suggesting the mtdsRNAs are moved to stress granules.

The presence mtdsRNAs in the cytosol mimics a viral invasion and triggers an IFN-1 response (Dhir et al, 2018). Thus, measuring the IFN-1 response is a more specific method to assess the IFN-1 response than quantitating the amount of mtdsRNA that localizes to the cytosol versus the mitochondria. We used qRT–PCR to quantitate the expression of a set of genes for the IFN response including *IFNB1* for IFN-β and the IFN-stimulated genes (ISGs) *IFI44* for antiviral activity and *ISG15* for signaling (Dzimianski et al, 2019). PNPase KD but not SUV3 KD resulted in the increased expression of the *IFNB1* and ISGs, indicating that the mtdsRNA indeed reaches the cytosol to induce the IFN-1 response (Fig 2C). Because MDA5 specifically binds to mtdsRNA (Dhir et al, 2018), we investigated RNA levels of *IFIH1* (gene for MDA5) with qRT–PCR 72 h after various RNAi treatments (Fig 2D). When PNPase was knocked down but not SUV3 or SUV3 and PNPase, *IFIH1* transcript levels increased markedly. Because MDA5 response with PNPase KD seemed very specific, we used *IFIH1* transcript analysis for subsequent studies. Thus, the IFN-1 pathway is specifically activated when PNPase is knocked down, confirming localization of mtdsRNA to the cytosol.

### PNPase functions in the intermembrane space (IMS) and the matrix

**Figure S5.**
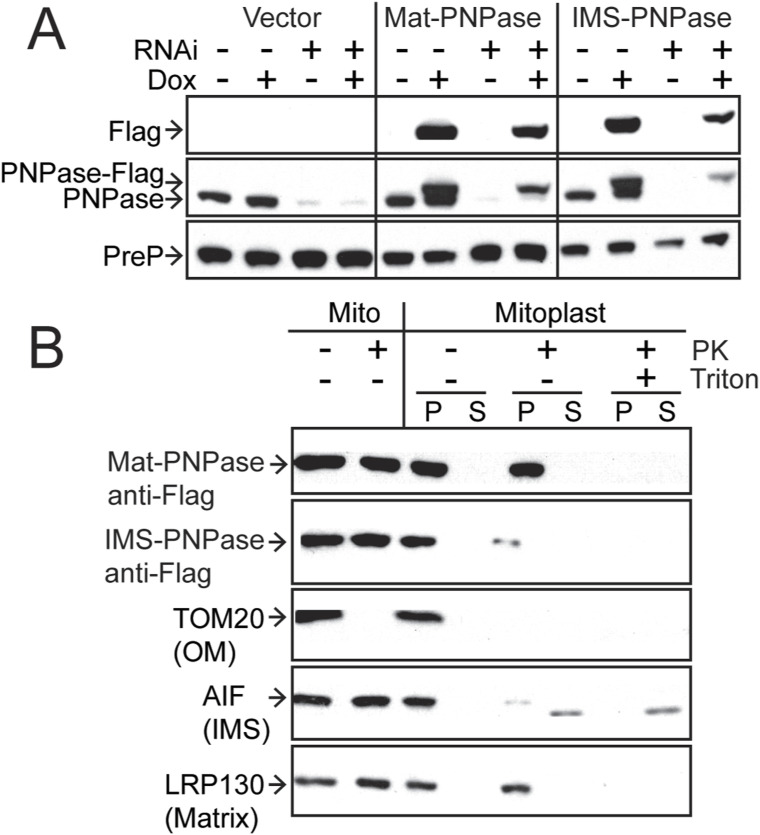
Characterization of Mat-PNPase and IMS-PNPase constructs confirms correct targeting. **(A)** As a control for Fig 3, the expression and localization of Mat-PNPase and IMS-PNPase constructs were confirmed by Western analysis. The constructs contained a C-terminal FLAG tag that was detected with an anti-FLAG antibody. Mitochondria were purified from HeLa cells, and half was subjected to proteinase K (PK) treatment followed by immunoblotting with antibodies against FLAG, TOMM20 (outer membrane), AIF (intermembrane space), and LRP130 (matrix). An aliquot of mitochondria was further treated to osmotic shock by dilution to 0.25 mM sucrose followed by PK treatment in the absence or presence of Triton X-100. **(B)**. HeLa cell lines were transformed with the empty vector, Mat-PNPase, or IMS-PNPase, and expression was induced with 500 ng/ml doxycycline. Simultaneously, endogenous PNPase was knocked down with RNAi. Antibodies against FLAG, PNPase, and PREP (loading control) were used to assess expression.

**Figure 3.**
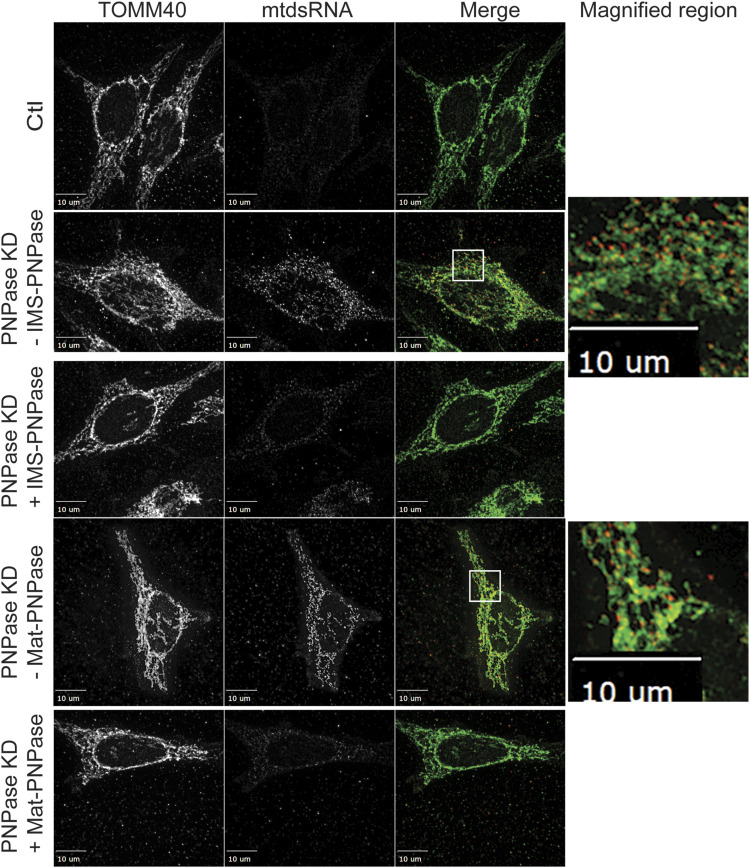
PNPase targeted to the IMS or matrix rescues PNPase KD. PNPase constructs were generated in which the native MTS was replaced with an IMS (IMS-PNPase) or matrix (Mat-PNPase) targeting sequence, and the constructs were integrated into a HeLa T-Rex Flp-In cell line; expression was induced with doxycycline. In a series of experiments, the expression of IMS-PNPase or Mat-PNPase was induced (+) with 500 ng/ml doxycycline or not induced (−) followed by PNPase KD. After 60 h, cells were fixed for imaging. The mtdsRNA (red) and mitochondria (green) were detected as in Fig 1. As a control (Ctl), the cells were treated with a scrambled RNAi construct. The bar indicates 10 μm. The right panel contains a magnified region from the merged image (marked with a white box) for conditions in which mtdsRNA was detected (*n* = 5).

Published studies report that PNPase localizes to the IMS and matrix (Chen et al, 2006; Rainey et al, 2006; Borowski et al, 2013). To determine the compartment in which PNPase functions, we replaced the endogenous mitochondrial targeting sequence (MTS), which consists of amino acids 1–37. To target to the IMS, a construct (designated IMS-PNPase) with the MTS (amino acids 1–68) from serine β-lactamase–like protein LACTB was generated (Polianskyte et al, 2009; Rhee et al, 2013). To target to the matrix, a construct (designated Mat-PNPase) with the MTS (amino acids 1–69) of *N. crassa* F_o_-ATPase subunit 9 was generated (Pfanner et al, 1987). The constructs were integrated into a HeLa T-Rex FLP-In cell line; expression was induced with 500 ng/ml doxycycline, and endogenous PNPase was simultaneously silenced (Fig S5A). Mat-PNPase and IMS-PNPase contained a C-terminal FLAG tag for detection, and the empty vector was also included as a control. Experiments in which the mitochondrial outer membrane was ruptured by incubation in hypotonic buffer but the inner membrane remained intact indicated that the constructs were targeted correctly (Fig S5B). Abundant mtdsRNAs (marked by red fluorescence) in the cytosol were detected upon knockdown of endogenous PNPase (Fig 3). However, induction of PNPase targeted to the matrix or IMS resulted in markedly reduced mtdsRNA puncta (Fig 3). Taken together, these results indicate that PNPase surprisingly functions in both the IMS and matrix.

### Outer membrane components VDAC and BAK/BAX mediate transport of mtdsRNAs

**Figure 4.**
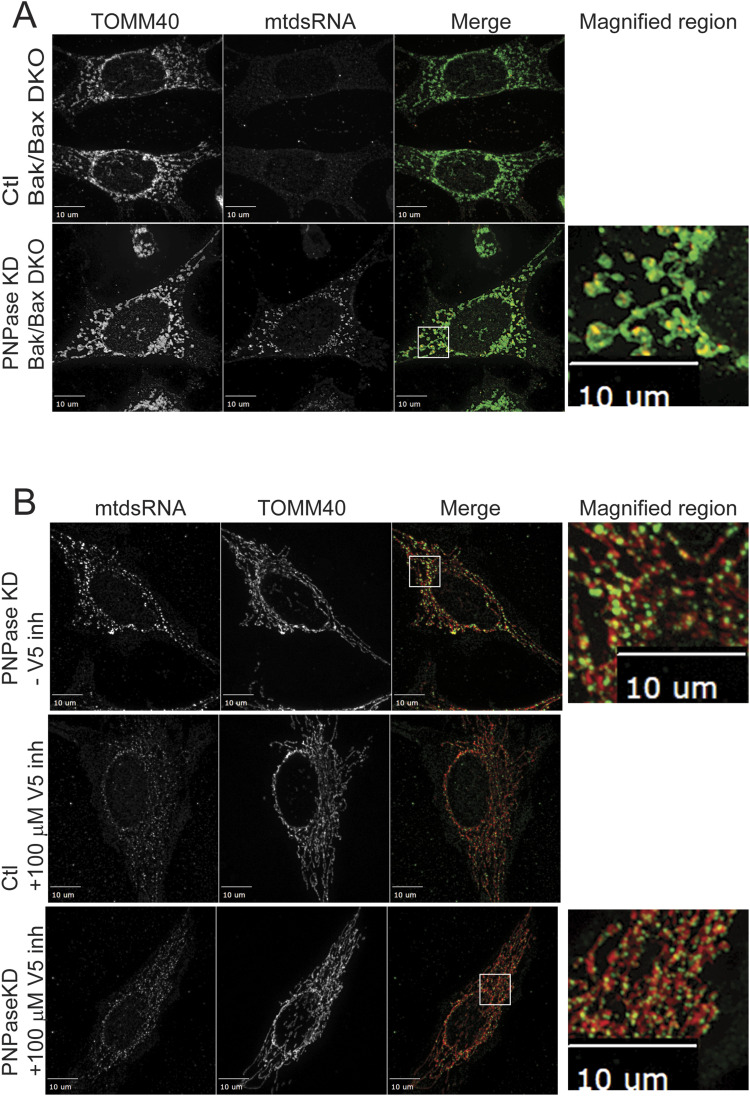
BAK and BAX are required for mtdsRNA release to the cytosol. **(A)** BAK/BAX double-knockout MEF cell line was used for PNPase KD as in Fig 1. Mitochondria (green) were marked with anti-TOMM40, mtdsRNA (red) was marked with anti-J2 antibody, and the images were merged. The right panel contains a magnified region from the merged image (marked with a white box) for conditions in which mtdsRNA was detected (*n* = 4). **(B)** HeLa cells were treated with 100 μM BAX inhibitor peptide V5, and PNPase was knocked down as in Fig 1. Mitochondria (green) were marked with anti-TOMM40, and mtdsRNA (red) was marked with anti-J2. The images were merged. As a control (Ctl), the cells were treated with a scrambled RNAi construct. The bar indicates 10 μm (*n* = 4).

In collaborative studies, we previously showed that BAK or BAX KD in combination with PNPase KD blocked the IFN-1 response (Dhir et al, 2018), suggesting that mtdsRNAs were not exported from mitochondria to the cytosol. Thus, we investigated the localization of mtdsRNA in the MEF cell line in which BAX and BAK were both knocked out (Bak/Bak DKO) (Takeuchi et al, 2005). RNAi constructs specific for mouse PNPase or a non-targeting RNAi construct (Ctl) were used for KD. The mtdsRNA (red) localization was determined by immunofluorescence, and mitochondria were marked with an antibody against TOMM40 (green) (Fig 4A). In WT MEFs, mtdsRNAs localized to the cytosol when PNPase was deleted (data not shown). However, in the MEFS that lacked BAK and BAX with PNPase KD, mtdsRNAs co-localized with mitochondria (PCC = 0.79) and were not released into large cytosolic puncta (Fig 4A). Instead, the mitochondrial morphology seems to be altered in that an enlarged mitochondrial network surrounds the mtdsRNA.

The BAX inhibitor peptide V5, which blocks BAX translocation to mitochondria (Gomez et al, 2007), was also used to determine whether mtdsRNA export was attenuated (Fig 4B). HeLa cells were treated with 100 μM V5 peptide (Gomez et al, 2007), and PNPase was simultaneously knocked down followed by immunofluorescence microscopy. Whereas mtdsRNA (green) localized to the cytosol in PNPase KD (PCC = 0.50), the addition of V5 peptide resulted in mtdsRNA that showed increased co-localization with mitochondria (red, PCC = 0.74). Taken together, BAK and BAX are important for mtdsRNA localization to the cytosol.

**Figure 5.**
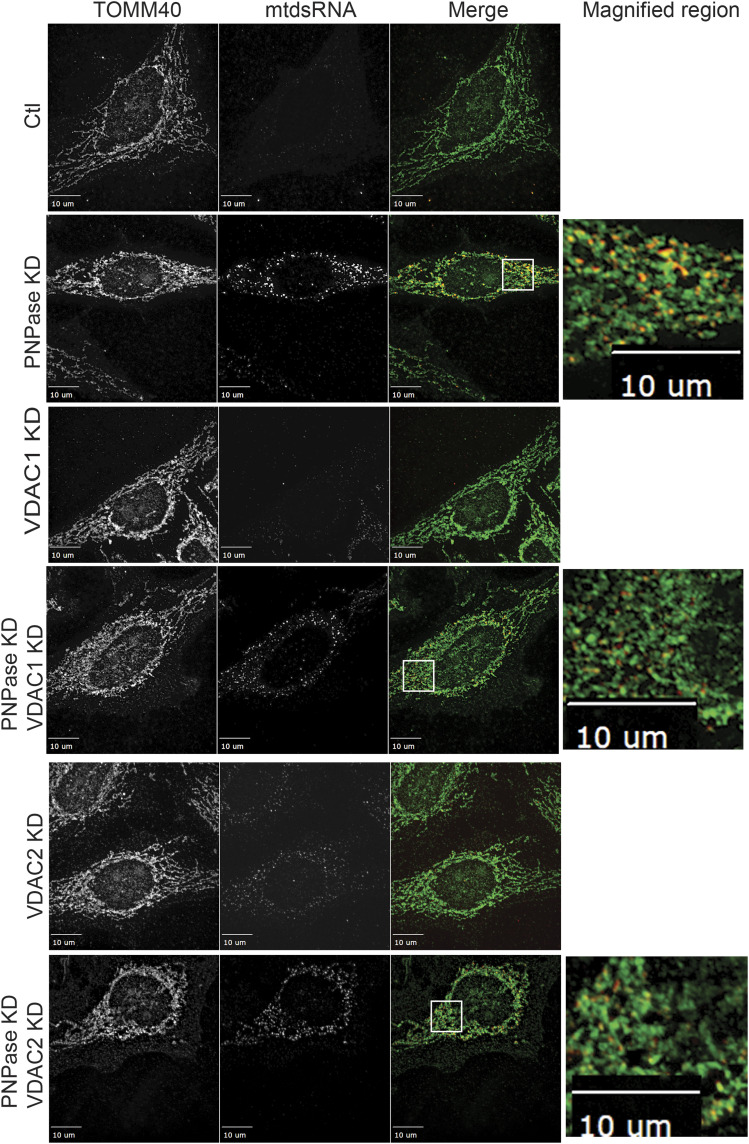
Knockdown or inhibition of VDAC1 or VDAC2 prevents mtdsRNA release to the cytosol. The indicated knockdown combinations for PNPase, VDAC1, and VDAC2 were generated in HeLa cells as in Fig 1. The mtdsRNAs (green) and mitochondria (red) were detected by immunofluorescence with the J2 antibody and anti-TOMM40, respectively. The merged images are presented in the right column. The control (Ctl) is a scrambled RNAi construct. The bar indicates 10 μm (*n* = 6).

Because VDAC mediates the import of RNAs into mitochondria, as well as the export of mtDNA from mitochondria (Kim et al, 2019), we also considered it as a candidate for mtdsRNA export. Of the three VDAC genes, VDAC1 is expressed most abundantly, followed by VDAC2, and finally, VDAC3 is least expressed (Yamamoto et al, 2006). VDAC2 through association with BAK inhibits apoptosis (Cheng et al, 2003), suggesting the VDAC2 may have a unique function. We investigated a combination of VDAC1 or VDAC2 KD with PNPase KD (Fig 5). The mtdsRNA (green) localized to the cytosol when PNPase alone was knocked down (PCC = 0.51), but KD combinations of VDAC1 (PCC = 0.81) or VDAC2 (PCC = 0.68) with PNPase resulted in mtdsRNA that markedly co-localized with mitochondria (red). Of note, the mitochondrial network seems more fragmented with VDAC KD. MDA5 expression also was not induced markedly when by VDAC1 or VDAC2 KD alone or in combination with PNPase KD (Fig 2D), supporting that mtdsRNA was not being exported to the cytosol.

**Figure S6.**
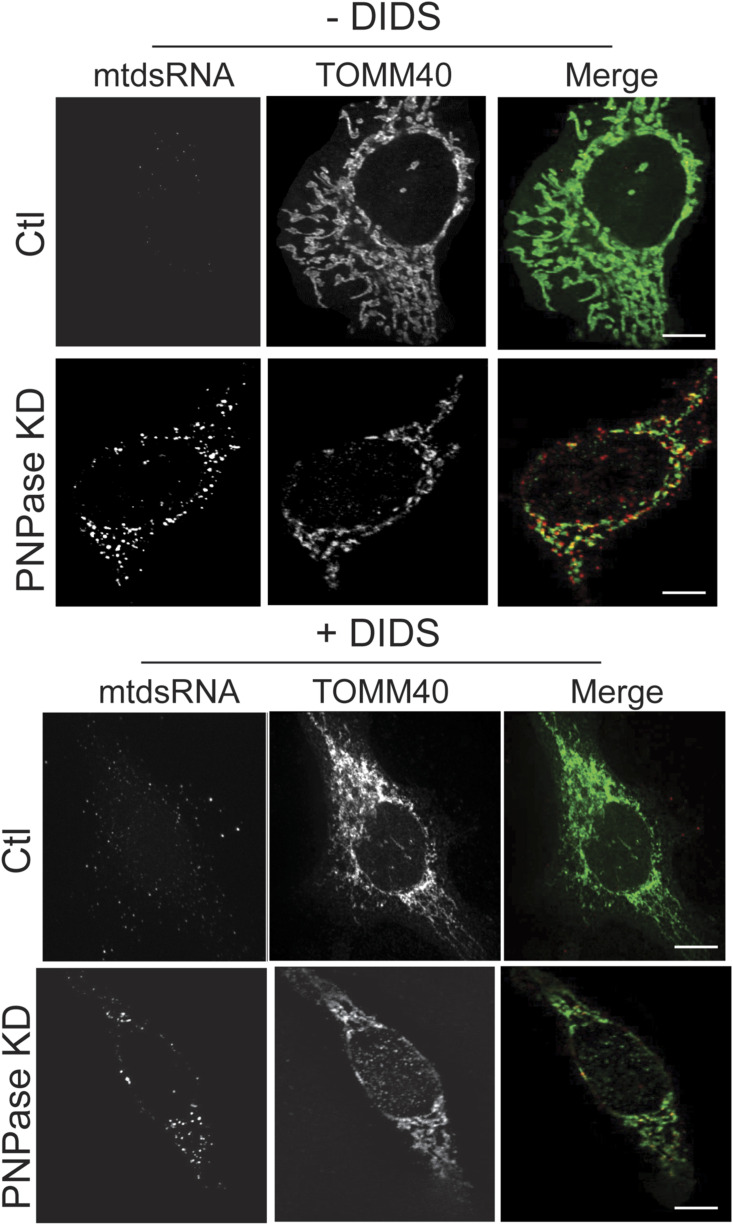
VDAC inhibitor DIDS prevents mtdsRNA export to the cytosol. As in Fig 5, HeLa cells were knocked down for PNPase, simultaneously treated with 50 μM DIDS, and then imaged at 60 h. Mitochondria (green) were marked with anti-TOMM40, and mtdsRNA (red) was marked with anti-J2. The images were merged. As a control (Ctl), the cells were treated with a scrambled RNAi construct for PNPase.

To complement the VDAC knockdown studies, we used the chemical 4,4′-diisothiocyanatostilbene-2,2′-disulfonate (DIDS) that inhibits the VDAC channel activity (Ben-Hail & Shoshan-Barmatz, 2016). DIDS is routinely used as an inhibitor for VDAC by blocking the channel and/or altering oligomerization (Ben-Hail & Shoshan-Barmatz, 2016). A titration curve with DIDS was conducted over 72 h, and a concentration of 50 μM, which did not inhibit cell growth or viability and was similar to the published literature (Kim et al, 2019), was selected. PNPase KD or the control RNAi construct was added to cells, and 24 h post-transfection, 50 μM DIDS was added. At 72 h post-transfection, cells were imaged for mtdsRNA and mitochondria (Fig S6). In cells treated with DIDS that lacked PNPase, mtdsRNA (red) co-localized to mitochondria (green). In contrast, mtdsRNA showed less co-localization with mitochondria as expected when cells were not treated with DIDS. Thus, treatment with DIDS and knockdown of VDAC resulted in mtdsRNAs remaining in mitochondria. In sum, outer membrane components VDAC, BAK, and BAX seem to play a role in mtdsRNA export to the cytosol.

### Prohibitin 1 and 2 are required for mtdsRNA export

**Figure S7.**
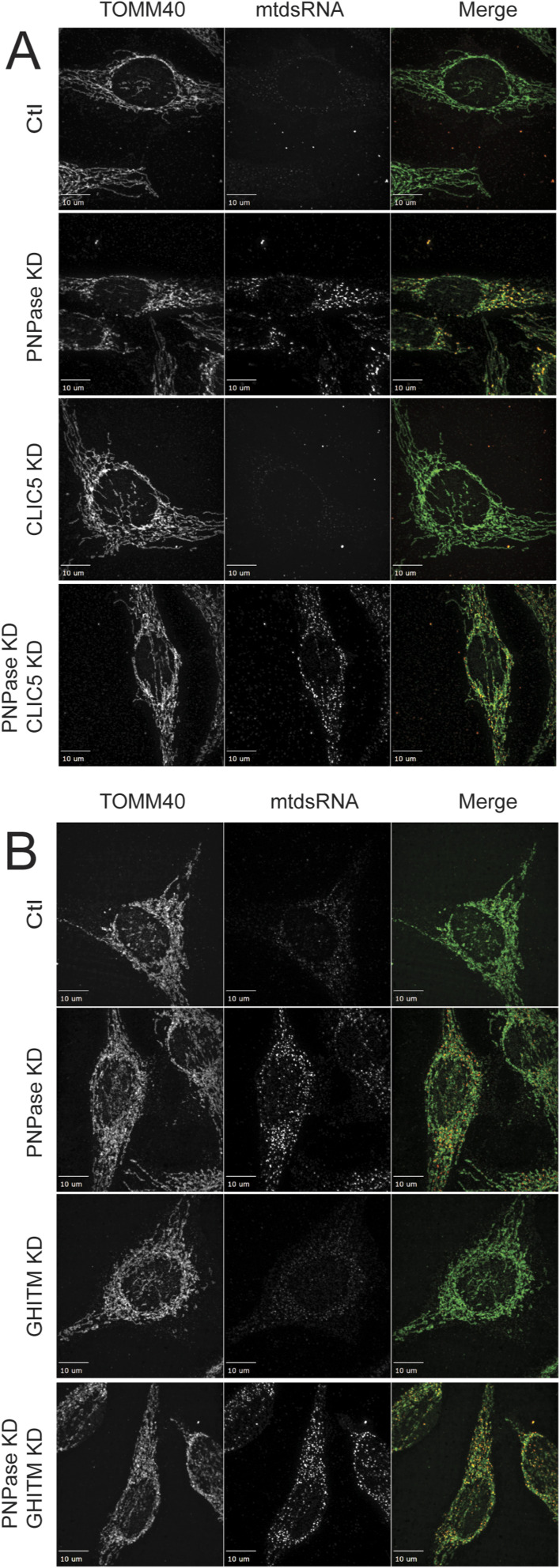
Knockdown of inner membrane proteins CLIC5 or GHITM does not block export of mtdsRNA to the cytosol. **(A)** As in Fig 5, the inner membrane protein CLIC5 and PNPase were knocked down by RNAi and in HeLa cells. The mtdsRNAs (red) and mitochondria (green) were detected by immunofluorescence with the J2 antibody and anti-TOMM40, respectively. The merged images are presented in the right column. The control (Ctl) is a scrambled RNAi construct for PNPase. The bar indicates 10 μm (*n* = 3). **(A, B)** As in “(A)” with GHITM.

**Figure 6.**
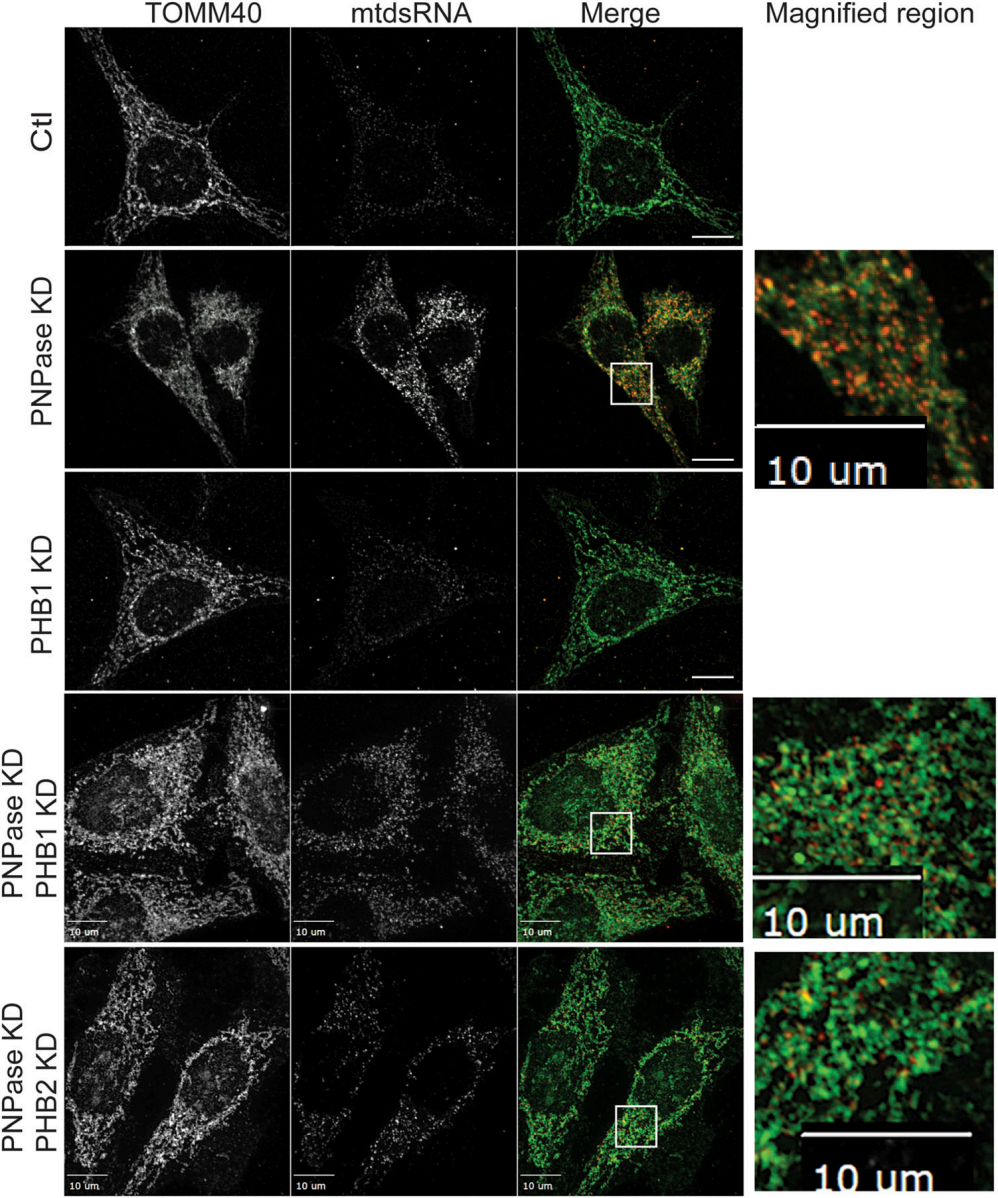
Knockdown or inhibition of PHB1 or PHB2 prevents mtdsRNA release to the cytosol. The indicated knockdown combinations for PNPase, PHB1, and PHB2 were generated in HeLa cells as in Fig 1. The mtdsRNAs (red) and mitochondria (green) were detected by immunofluorescence with the J2 antibody and anti-TOMM40, respectively. The merged images are presented in the right column. The control (Ctl) is a scrambled RNAi construct for PNPase. The bar indicates 10 μm (*n* = 5).

The mechanism by which mtDNA or mtdsRNA crosses the mitochondrial inner membrane is not clear. Published studies suggest that mtDNA might be released by herniation of the inner membrane (McArthur et al, 2018; Riley et al, 2018) or by mitochondrial-derived vesicles (Cadete et al, 2016). Given that mitochondrial physiology was not severely compromised in a conditional mouse model that lacked PNPase in the liver (Wang et al, 2010), we reasoned that the inner membrane might have a regulated mechanism for mtdsRNA release that does not severely compromise overall mitochondrial function. In contrast, the release of mtDNA from mitochondria might be expected to result in apoptosis, because mtDNA is essential for viability. From mass spectrometry experiments and the published literature, we considered (1) the anionic inner membrane channel CLIC5, (2) the BAK/BAX-interacting inner membrane protein GHITM, and (3) inner membrane proteins that may regulate inner membrane dynamics, prohibitin 1 and 2 (PHB1/2), as potential candidates. Although the interactions with PNPase in mass spectrometry studies were not robust (Yoshinaka et al, 2019), PHB1/2 and CLIC5 are also implicated in the IFN-1 response (Langlais et al, 2016; Yoshinaka et al, 2019; Refolo et al, 2020), which is similar to PNPase. The KD of CLIC5 or GHITM in combination with PNPase KD did not block the release of mtdsRNAs (red) from mitochondria (green) (Fig S7A and B), thereby eliminating them as potential candidates. Because PHB1 and PHB2 co-assemble, the proteins were knocked down individually (Nijtmans et al, 2000; Tatsuta et al, 2005). When PHB1 (Fig 6) or PHB2 (data not shown) was knocked down singly, mtdsRNA was not detected, in contrast to KD of PNPase (PCC = 0.42). The KD of PHB1 (PCC = 0.74) or PHB2 (PCC = 0.73) in combination with PNPase KD blocked the release of mtdsRNAs (red) to the cytosol, and the mtdsRNA remained in mitochondria (green) (Fig 6). In addition, the abundance of mtdsRNAs seemed to be somewhat decreased. To confirm that the mtdsRNA did not induce the IFN-1 response, MDA5 expression was not markedly elevated when PNPase was knocked down in combination with PHB1 or PHB2 (Fig 2D).

**Figure S8.**
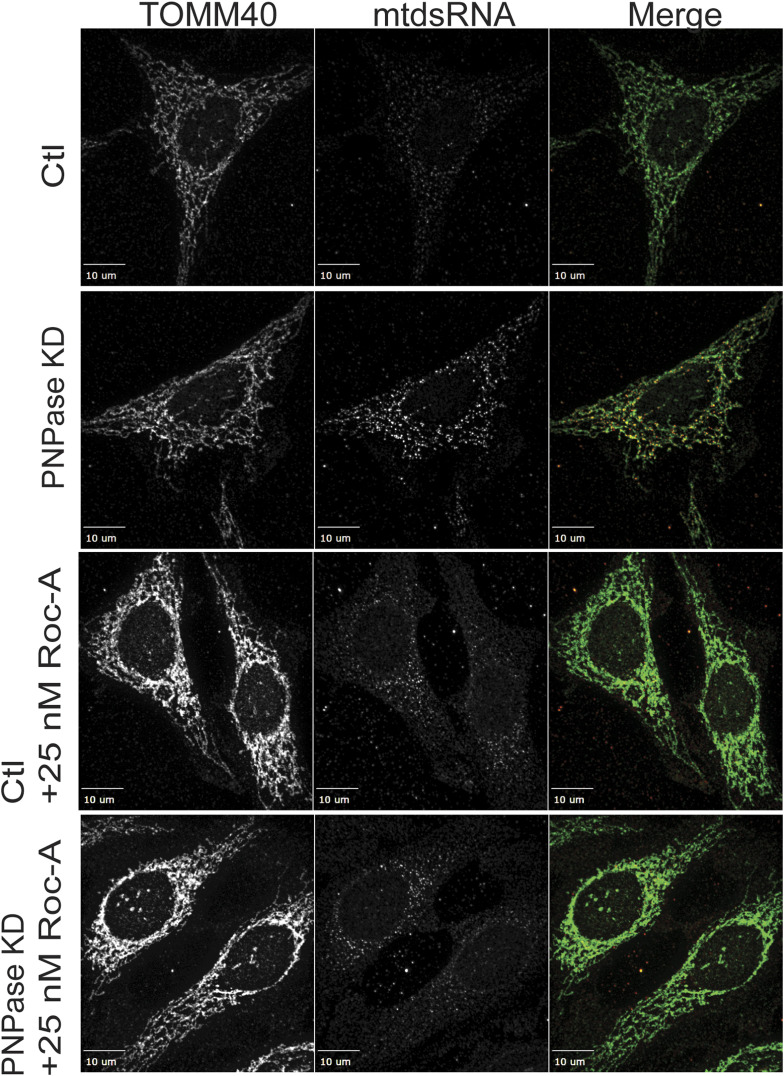
Treatment with the PHB inhibitor, rocaglamide, prevents mtdsRNA release to the cytosol. As in Fig 6, HeLa cells were knocked down for PNPase and simultaneously treated with 25 nM rocaglamide (Roc-A) followed by imaging at 60 h. Mitochondria (green) were marked with anti-TOMM40, and mtdsRNA (red) was marked with anti-J2. The images were merged. As a control (Ctl), the cells were treated with a scrambled RNAi construct. The bar indicates 10 μm (*n* = 5).

We also tested the PHB1/2 inhibitor, rocaglamide (Roc-A), which has been shown to inhibit PHB1/2 assembly (Polier et al, 2012). Roc-A was titrated over a 72-h period, and a concentration of 25–50 nM did not seem to impair cell growth. Cells were knocked down with PNPase or the control RNAi construct, and 25 nM Roc-A was added after 24 h followed by imaging at 72 h (Fig S8). The mtdsRNA (red) pool again co-localized with mitochondria (green), indicating that PHB1/2 seem to play a role in the export of mtdsRNAs to the cytosol. Thus, a candidate for release of mtdsRNAs from mitochondria is the PHB1/2 complex.

### Cytosolic mtdsRNAs are detected in a subset of lung cancers

**Figure S9.**
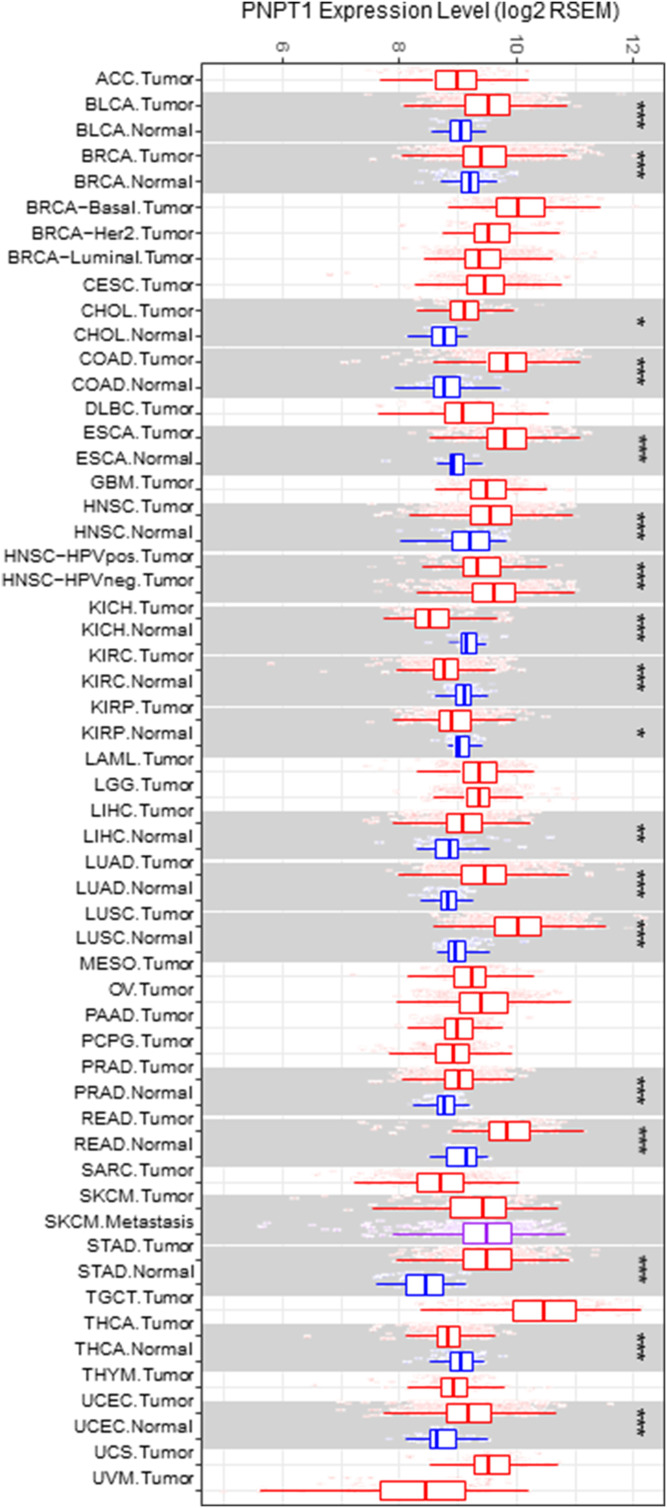
Analysis of PNPT1 expression in various tumors indicates PNPase may be up-regulated. The expression level of PNPT1 was analyzed from data in The Cancer Genome Atlas project using Tumor Immune Estimation Resource (TIMER; https://cistrome.shinyapps.io/timer/) (PMID: 29092952) in a variety of tumors (shown in red), including lung adenocarcinoma (LUAD) and lung squamous cell carcinoma (LUSC), and normal controls (shown in blue), when available. Statistical significance was calculated by the Wilcoxon test. Additional abbreviations include ACC, adrenocortical carcinoma; BLCA, bladder urothelial carcinoma; BRCA, breast invasive carcinoma; CESC, cervical and endocervical carcinoma; CHOL, cholangiocarcinoma; COAD, colon adenocarcinoma; DLBC, diffuse large B-cell carcinoma; ESCA, esophageal carcinoma; GBM, glioblastoma multiforme; HNSCC, head and neck squamous cell carcinoma; KICH, kidney chromophobe; KIRC, kidney renal clear cell carcinoma; KIRP, kidney renal papillary cell carcinoma; LAML, acute myeloid leukemia; LGG, brain lower grade glioma; LIHC, liver hepatocellular carcinoma; MESO, mesothelioma; OV, ovarian serous cystadenocarcinoma; PAAD, pancreatic adenocarcinoma; PCPG, pheochromocytoma and paraganglioma; PRAD, prostate adenocarcinoma; READ, rectum adenocarcinoma; SARC, sarcoma; SKMC, skin cutaneous melanoma; STAD, stomach adenocarcinoma; TGCT, testicular germ cell tumors; THCA, thyroid carcinoma; THYM, thymoma; UCEC, uterine corpus endometrial carcinoma; UCS, uterine carcinosarcoma; UVM, uveal melanoma. One asterisk represents *P* < 0.05, two asterisks represent *P* < 0.01, and three asterisks represent *P* < 0.001.

Release of mtDNAs has been reported in different cancer cell lines (Wu et al, 2021), particularly in non–small-cell lung cancer (NSCLC) cell lines where mtDNA release induces the STING signaling pathway (Kitajima et al, 2019). In addition, Roc-A treatment was shown to inhibit proliferation, migration, and anchorage-independent growth in a subset of *KRAS*-mutated NSCLC cell lines (Yurugi et al, 2017), suggesting mtdsRNA may be an important DAMP. In previous studies, we have characterized NSCLC cell lines and found they can be separated by preference for oxidative (OXPHOS^HI^) versus glycolytic (OXPHOS^LOW^) metabolism (Momcilovic et al, 2019). We reasoned that, in addition to mtDNA export, mtdsRNAs might also be exported in a subset of NSCLCs, particularly those that are glycolytic (Shimada et al, 2018). Analysis of The Cancer Genome Atlas (TCGA) database showed that PNPase (*PNPT1*) gene expression was up-regulated significantly in a subset of lung adenocarcinoma (LUAD) and lung squamous cell carcinoma (LUSC) lines (Fig S9) (Momcilovic et al, 2018). In addition, PNPase expression was up-regulated in numerous tumor types, compared with controls (Fig S9). Because PNPase is an IFN-1–induced gene (Leszczyniecka et al, 2003), increased PNPT1 expression in tumors may reflect those that have an increased IFN-1 response.

**Figure 7.**
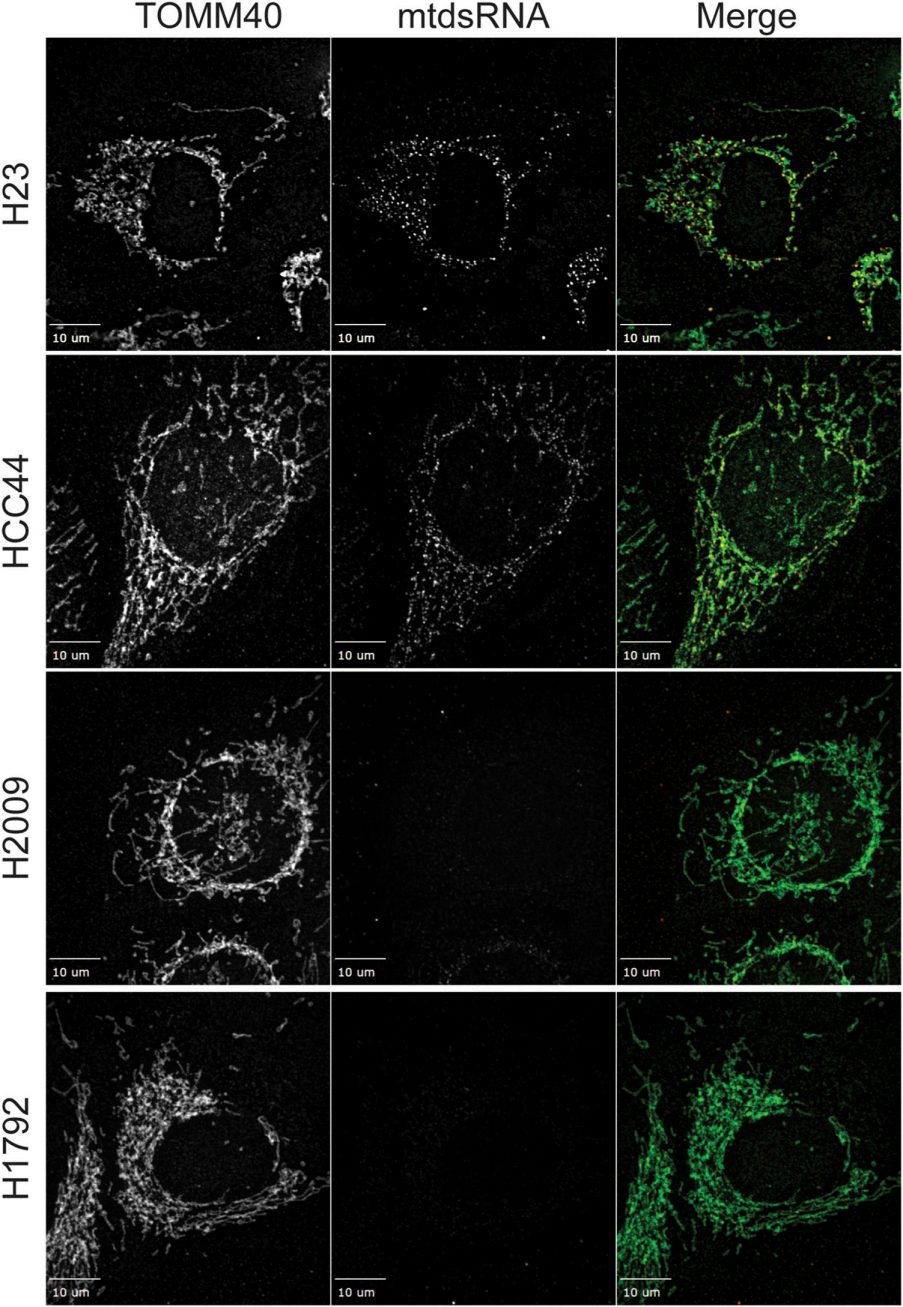
Subset of NSCLC cell lines have mtdsRNA localized to the cytosol. The NSCLC cell lines, H23, HCC44, H2009, and H1792, were grown on coverslips and fixed for immunofluorescence. Mitochondria (green) and mtdsRNA (red) were detected as in Fig 1. The bar indicates 10 μm (*n* = 3).

With well-characterized NSCLC lines, we investigated whether mtdsRNAs were detected using immunofluorescence. Barbie and colleagues previously reported in the LUAD cell line H1792 (co-mutations in *KRAS* and *TP53*, designated KP) that mtDNA was not detected in the cytosol (Kitajima et al, 2019); this cell line has an OXPHOS^HI^ metabolic profile (Chen et al, 2019). Similarly, immunofluorescence studies showed that mtdsRNAs were not detected (Fig 7). In addition, the cell line H2009, which has an OXPHOS^HI^ metabolic profile as well, lacked mtdsRNA. In contrast, abundant mtdsRNAs were present in LUAD cell lines, HCC44 and H23, with co-mutations in *KRAS* and *STK11*/*LKB1* (designated KL) (Fig 7). These cell lines have an OXPHOS^LOW^, glycolytic, metabolic profile (Meijer et al, 2018; Majem et al, 2020). In the cell line H23, a cohort of the mtdsRNA seemed to localize to the cytosol in larger puncta when compared to the mtdsRNA cohort in HCC44 cells. Thus, in a subset of NSCLC cell lines, mtdsRNA may be exploited as a DAMP for signaling mitochondrial stress.

### mtdsRNAs in a subset of NSCLC lines are detected by sequencing

**Figure 8.**
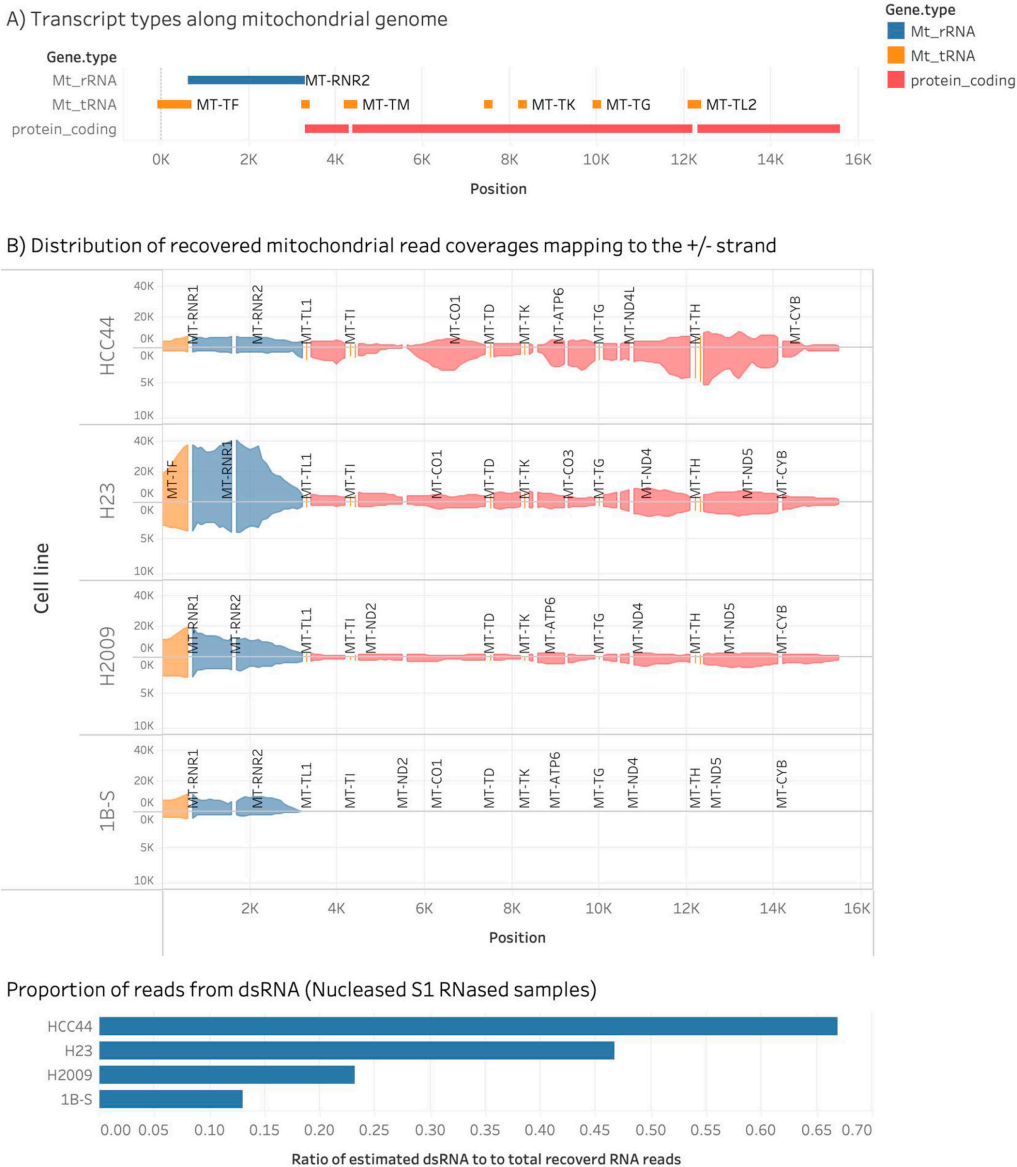
Sequencing of the dsRNA in H23 and HCC44 cell lines confirms that the dsRNA is from mitochondria. RNA was purified from the H23, HCC44, and H2009 cell lines, and a negative control cell (1B-S) line, and dsRNA was prepared for sequencing. The total content and fraction of mitochondrial double-stranded reads were determined by sliding a window across the aligned read files and returning the coverage of reads mapping to the ± strand for each window. **(A)** Schematic of the mitochondrial genome, including coding regions, ribosomal RNAs, and tRNAs. **(B)** Distribution of aligned reads to the mitochondrial genome. The x-axis represents mitochondrial DNA coordinates (3k to 16k). The y-axis indicates the number of reads that overlap each sliding window for each of the four samples (HCC44, H23, H2009, and 1B-S). Positive and negative reads are represented, respectively, by regions above and below the baseline. The lower panel displays the estimated proportion of dsRNA reads per sample.

To confirm that the dsRNAs are derived from mitochondria, we sequenced mtdsRNA from the NSCLC cell lines (Dhir et al, 2018). Briefly, a cell lysate was treated with DNase I to degrade DNA, then treated with S1 RNase to remove single-stranded RNA. To the remaining dsRNA, adapters were ligated followed by strand-specific RNA sequencing. The reads were aligned against the mitochondrial genome (Fig 8A). As a negative control, a cell line (1B-S) in which mtdsRNA was not detected by immunofluorescence was included (Fig 8B); a small amount of mitochondrial ribosomal DNA was detected, which probably formed secondary structures (Fig 8B). Similarly, a low amount of mtdsRNA was detected in the H2009 cell line. In contrast, a large amount of mtdsRNA was detected along the entire mitochondrial chromosome in the H23 and HCC44 cell lines. Interestingly, the H23 cell line has large amounts of rRNA, whereas the HCC44 cell line had greater reads in the coding region. Thus, mtdsRNA was most abundant in cell lines HCC44 and H23 as shown in the bottom panel (Fig 8B), confirming that mitochondria are the source of the dsRNA. Studies in NSCLC cell lines support that mtdsRNA may be a novel DAMP in lung cancer, supporting a physiologic role of this pathway in cancer.

## Discussion

Because patients with PNPase mutations and mtdsRNA accumulation in the cytosol can survive until late in life (Rius et al, 2019), mitochondrial function was not strongly compromised. Indeed, a mouse model that lacks PNPase in the liver is healthy (Wang et al, 2010), but shows a 50% reduction in OXPHOS activity in the liver. This suggests that mitochondria have a physiologic pathway for mtdsRNA release to the cytosol. Here, we begin to outline a pathway for the export of mtdsRNAs from mitochondria to the cytosol where they up-regulate the IFN-1 response. The J2 antibody in combination with sequencing has been an important tool for detecting and characterizing mtdsRNA. Statistical measures to assess localization to the cytosol versus the mitochondrion are difficult to use in assessing whether the mtdsRNA has trafficked to the cytosol. Instead, the best measure for induction of the IFN-1 response seems to be the analysis of IFIH1 transcripts by qRT-PCR analysis. Because the mtdsRNA, upon PNPase KD, is rapidly transferred to puncta that contain TIA-1, scant amounts of mtdsRNA may be required in the cytosol to induce the IFN-1 response. Surprisingly, mtdsRNA from SUV3 KD does not induce the IFN-1 response; perhaps the mtdsRNA is actively held within mitochondria. We propose that mtdsRNAs can be considered as a DAMP to signal mitochondrial stress in a subset of cancers and, potentially, other pathogenic states. Mechanistically, many details remain to be characterized. However, this study supports that mtdsRNA might be a useful marker for cancer diagnostics and the mtdsRNA export pathway might provide new targets for developing therapeutic strategies in treating cancer.

### PNPase seems to function in both the matrix and IMS with PHB1/2

PNPase has been placed in numerous compartments. Originally, PNPase was identified as a cytosolic protein that played a role in terminal differentiation and cellular senescence (Leszczyniecka et al, 2002). However, PNPase has an N-terminal targeting sequence that directs its import into the mitochondrial IMS in a pathway that depends on Yme1 in vitro (Rainey et al, 2006). Submitochondrial fractionation also showed that PNPase resided primarily in the IMS (Chen et al, 2006). Additional studies suggested that a fraction resides in the matrix and binds to SUV3 (Borowski et al, 2013), which aligns with its role in RNA degradation. Finally, PNPase has been reported to have a role in decay of cytosolic poly(A) RNAs when released during apoptosis (Liu et al, 2018), but late release of PNPase does likely not support degradation of cytosolic RNA as its primary function (Chen et al, 2006).

Given that loss of PNPase results in the export of mtdsRNAs to the cytosol, we took advantage of this activity to target PNPase to the IMS or matrix by appending a different targeting sequence. The IMS and matrix targeting sequences localized PNPase to the IMS or matrix, respectively. When endogenous PNPase was knocked down by RNAi in combination with the induced expression of the matrix- and IMS-forms of PNPase, the mtdsRNA levels were drastically reduced. These results support that PNPase seems to function in both the IMS and matrix. Mitochondrial proteins that are dual-localized to the matrix and IMS are rare. Potentially, PNPase is part of a larger export complex or channel that may contain PHB1/2, allowing PNPase to function on either side of the IM.

We used a candidate approach to determine potential components that function at the IM to release mtdsRNAs. Our criteria were IM proteins that had potential channel properties and induced the IFN-1 response, as well as interactions with PNPase (albeit weak). Previously, we have tried to identify partner proteins for PNPase but have not identified robust partners, and PNPase in yeast migrates in the same-sized complex as in mouse mitochondria at the inner membrane (Chen et al, 2006). Thus, PNPase seemed to lack partner proteins and may instead transiently associate with other partners.

We considered GHITM and CLIC5, but mtdsRNAs were exported when PNPase was knocked down. Instead, reduction of PHB1/2 by knockdown or inhibition with the small molecule inhibitor Roc-A seemed to prevent the export of mtdsRNAs to the cytosol. The abundance of mtdsRNAs also seemed to be somewhat decreased. Given that PHB1/2 and PNPase expression is increased by the IFN-1 response (Yoshinaka et al, 2019), they seem to align well for export of mtdsRNA from the IM. Early cryo-EM studies showed that PHB1/2 can form a large ring (Tatsuta et al, 2005), but its structure in the mitochondrial inner membrane is not known. PHB1/2 is structurally related to the bacterial HflKC complex that binds to the AAA protease FtsH (Qiao et al, 2022). A cryo-EM structural analysis showed that 12 copies of HflK and HflC form a large cage in the membrane and four FtsH hexamers with periplasmic domains and transmembrane helices enclosed inside the cage and cytoplasmic domains situated at the base of the cage (Qiao et al, 2022). This large complex has a pore that is the size of the nascent polypeptide exit channel of the ribosome; however, given the intricacy, the complex could be dynamic in accommodating the transport of cargos such as mtdsRNA. Whether the mitochondrial AAA proteases YmeL1 and Afg3L2 function with PHB1/2 in export of mtdsRNAs is not known. Interestingly, YmeL1 has a role in mtDNA escape (Thorsness et al, 1993; Sprenger et al, 2021). Alternatively, the heptad repeat region of PHB2 has been suggested to form a scaffold with CLPB, ATAD3A, and AKAP1 and invoke the immune response from the mitochondrial signalosome (Yoshinaka et al, 2019). It is also likely that other proteins may participate and different pathways may exist for mtdsRNA export. A recent work by Wredenberg and colleagues showed that mtdsRNAs escape from mitochondria under conditions in which mitochondrial mRNA polyadenylation or degradation was disrupted in the fly model (Pajak et al, 2019). Finally, protein kinase RNA-activated recognizes short mtdsRNAs and can move in and out of mitochondria (Kim et al, 2018).

### Mitochondrial outer membrane permeabilization is important for release of mtdsRNAs at the outer membrane

The release of mtDNA from mitochondria at the outer membrane via mitochondrial outer membrane permeabilization with subsequent activation of the cGAS/STING pathway has been well established (McArthur et al, 2018; Riley et al, 2018; Xian et al, 2022). Here, we show that VDAC and BAK/BAX also play a role in the release of mtdsRNAs. In MEF cells that are knocked out for both BAK and BAX, mtdsRNA co-localized with mitochondria. In addition, VDAC also seems to play a role. This may not be so surprising as mtDNA relies on the same proteins; however, the specific mechanism and similarities between mtDNA and mtdsRNA export are not known. A recent study suggests that the assembly rate of BAX and BAK in large channels is an important determinant of the kinetics of mtDNA release (Cosentino et al, 2022). The release of mtDNA is often associated with apoptosis (Cosentino et al, 2022); however, mtdsRNA release did not result in apoptosis, suggesting the export pathways may differ. VDAC may be an important player for differentiating between mtDNA and mtdsRNA. Alternatively, mtdsRNA may be released under low-stress conditions, with mtDNA being released under high-stress conditions when apoptosis is triggered.

### In cancer, mtdsRNA as a new DAMP

A recent publication by Barbie and colleagues (Kitajima et al, 2019) dissected the underlying mechanism of immune resistance in NSCLC cell lines and found that *LKB1* loss resulted in suppression of the IFN genes (STING) and insensitivity to cytoplasmic double-stranded DNA detection. They showed that mtDNA was released from mitochondria to the cytosol and activated STING and the innate immune response (Kitajima et al, 2019). Thus, mtDNA release from mitochondria represents an unexpected intrinsic tumor-sensing mechanism. We also find in a subset of NSCLC cell lines that mtdsRNA could be detected in LUADs characterized by an OXPHOS^LOW^, glycolytic, metabolic profile (Shackelford & Shaw, 2009; Shackelford et al, 2013; Momcilovic et al, 2019). We confirmed that the dsRNA was derived from the mitochondria by sequencing. Indeed, most of the mitochondrial coding regions seemed to be detected, with increases in certain parts of the mitochondrial genome. In addition, the distribution of mtdsRNA in mitochondria versus cytosol seemed to differ between lung cancer cell lines with KP and KL mutations, suggesting that NSCLC cells with loss-of-function mutations in the *LKB1 (STK11)* gene may have stronger activation of the IFN-1 pathway. Given that mtDNA release is likely associated with apoptosis and innate immune responses, mtdsRNA may represent a new marker for characterizing NSCLC types and other cancers. Moreover, this pathway might be targeted for therapeutic strategies. For example, Roc-A is a member of the flavagline family that has antitumor properties and sorafenib suppresses the both DNA- and RNA-sensing–mediated IFN-1 pathway in hepatocellular carcinoma (Huang et al, 2022). Thus, the pathway by which mtdsRNAs are generated represents a new pathway to characterize broadly in cancer. In tumors that have an IFN-1 signature, mtdsRNA may be an activator (Budhwani et al, 2018). Alternatively, in tumors that become resistant to IFN signaling through cGAS/STING (Benci et al, 2016; Jiang et al, 2020), activation of mtdsRNA release may provide a new pathway to reactivate the immune system, particularly if mtDNA release was an initial activator (Kitajima et al, 2019). Additional published studies support that mtdsRNA is detected in numerous tumors (Killarney et al, 2023).

## Materials and Methods

### Cell lines and culture media

HeLa (American Type Culture Collection [ATCC]), HeLa T-REx Flp-In (Life Technologies), MEF Bax Bak DKO SVO (ATCC), and NSCLC cell lines H23 (ATCC), HCC44 (DSMZ), H2009 (ATCC), and H1792 (ATCC) were maintained at 37°C in a humidified incubator with 5% CO_2_. Cells were grown in high-glucose DMEM supplemented with 10% FBS, 100 U/ml penicillin, and 100 μg/ml streptomycin, with the exception of the MEF cell line that was supplemented with 5% FBS. Transfections were performed using BioT transfection reagent (Bioland) or Lipofectamine 2000 (Thermo Fisher Scientific) according to the manufacturer’s instructions. All cell lines were routinely tested and confirmed to be free of mycoplasma using the MycoAlert mycoplasma detection kit (Lonza).

### Chemicals

Chemicals were dissolved in DMSO, stored at −80°C, and used at the indicated concentrations. DMSO at 0.1% served as the vehicle control. The following chemical inhibitors were used: Bax-inhibiting peptide V5 (#196810; Sigma-Aldrich), prohibitin-binding rocaglamide-A (#S7428; Selleck Chemicals), and VDAC inhibitor 2,2'-(1,2-ethenediyl)bis(5-isothiocyanato-benzenesulfonic acid) (DIDS) (#16125; Cayman Chemical).

### Constructs

Constructs to target PNPase specifically to the IMS (IMS-PNPase) or matrix (Mat-PNPase) were constructed by removing the MTS (amino acids 1–37) from PNPase and replacing it with the MTS (amino acids 1–68) from serine β-lactamase–like protein LACTB (IMS) (Polianskyte et al, 2009; Rhee et al, 2013) or the MTS (amino acids 1–69) of *N. crassa* F_o_-ATPase subunit 9 (Pfanner et al, 1987). IMS-PNPase and Mat-PNPase contained a C-terminal FLAG tag for detection. The constructs were cloned into pcDNA5/FRT/TO and then integrated into a HeLa T-Rex FLP-In cell line by transfecting cells with 1.8 μg pOG44 (Life Technologies) and 0.2 μg pcDNA5/FRT/TO IMS-PNPase or Mat-PNPase expression construct using BioT transfection reagent in a six-well plate. After 48 h, the cells were transferred to a 10-cm dish and stable cells were selected with 200 μg/ml hygromycin B (Wako Pure Chemical Industries). Stable colonies were selected and maintained in 100 μg/ml hygromycin B. IMS-PNPase and Mat-PNPase expression was induced with 500 ng/ml doxycycline for 48 h.

### RNAi knockdown and small molecule modulators

Silencer Select siRNAs as indicated in Table S1 were used for knocking down target genes. Briefly, HeLa cells were transfected with 20 nM gene-specific Silencer Select using Polyplus jetPRIME transfection reagent, and 48–96 h post-transfection, cells were harvested for knockdown confirmation and immunofluorescence studies. The knockdowns were confirmed by Western blot analysis. Bax-inhibiting peptide V5 (100 μM) was added simultaneously with an RNAi construct, whereas rocaglamide-A (25 nM) and DIDS (50 μM) were added 24 h post-RNAi transfection.

Table S1. siRNA construct information used in this study.

### Immunofluorescence

Cells were transferred to six-well plates including coverslips. After 24 h, cells were attached to coverslips and were fixed in 4% formaldehyde, then permeabilized in 0.25% Triton X-100. Subsequently, cells were blocked in 0.1% phosphate-buffered saline with 3% BSA and 0.1% Tween-20, followed by incubation with the primary antibody (Table S2) overnight. Cells were washed and incubated with the appropriate secondary antibodies for 1 h. The cells were washed and transferred to mounting media (Antifade GOLD reagent with DAPI; Thermo Fisher Scientific). A 3i Spinning Disk Confocal microscope (Marianas) consisting of CSU-X1 A1 Spinning Disk (Yokogawa) attached to a Zeiss Axio Observer 7 microscope (Zeiss) was used. Standard filter sets were used for imaging. The PCCs were calculated using the JACoP plugin on ImageJ.

Table S2. Antibodies used in this study.

### Western blotting

Cells were lysed in RIPA buffer (50 mM Tris–HCl, pH 7.5, 150 mM NaCl, 1% NP-40, 0.1% SDS, 0.5% sodium deoxycholate, 1 mM EDTA, 1 mM PMSF [phenylmethylsulfonyl fluoride]) for 30 min on ice. After centrifugation at 21,000*g* for 10 min, the protein concentration was measured with the BCA protein assay kit (Thermo Fisher Scientific). Equal amounts of protein extracts were separated by SDS–PAGE and transferred to Immobilon PVDF membranes (Millipore). Proteins were detected with the indicated antibodies (Table S2) followed by chemiluminescence and imaged on ChemiDoc MP Imager (Bio-Rad).

### Mitochondrial isolation from cultured cells

For large-scale mitochondrial isolation, cells were resuspended in homogenization buffer (20 mM Hepes, pH 7.4, 220 mM mannitol, 70 mM sucrose, 0.5 mM PMSF, 2.5 mM NaF, and 1 mM Na3VO4) and ruptured with 30 strokes in a Teflon-glass homogenizer. The homogenized cellular extract was centrifuged twice at 850*g* for 10 min to obtain the post-nuclear supernatant. Mitochondria were pelleted at 10,000*g* for 10 min and washed once in homogenization buffer. For small-scale mitochondrial isolation, cells were resuspended in homogenization buffer, if necessary supplemented with 10 mM NEM and 10 μM MG132, and lysed by passing the cell mix through a 25-gauge needle 20 times with a 1-ml syringe. The extract was centrifuged twice at 770*g* for 5 min, and the supernatant was pelleted at 10,000*g* for 10 min to isolate mitochondria. The mitochondria were washed once with homogenization buffer. The yield was determined with the BCA protein assay kit after lysing the mitochondria in 0.1% SDS.

### PNPT1 expression analysis in tumors

The gene expression data for PNPT1 from RNA sequencing and corresponding clinical information were retrieved from The Cancer Genome Atlas (https://portal.gdc.cancer.gov/). The expression of PNPT1 was analyzed as previously described (Momcilovic et al, 2018). The statistical tests were calculated using GraphPad Prism.

### Immunoprecipitation of dsRNA

Cells were lysed in NP-40 lysis buffer (catalog #: J60766) for 30 min on ice. Protein A/G beads (catalog #: 88803) were incubated with J2 antibody suspended in NP-40 lysis buffer for 1 h at room temperature. The cell lysates were then incubated with the beads for 2 h at 4°C. The beads were washed with a high-salt PBS buffer (1x PBS, 0.1% Tween, 1 M NaCl) followed by two washes with a low-salt PBS buffer (1x PBS, 0.1% Tween, 0.5 M NaCl). The dsRNA from the immunoprecipitation was eluted by treating with 1 mg/ml Proteinase K (catalog #: P8044-1G) in NP-40 lysis buffer.

### Sequencing of immunoprecipitated dsRNA with the Oxford Nanopore platform

The immunoprecipitated RNA with the J2 antibody was extracted with phenol–chloroform and then precipitated using ethanol. Sequencing libraries were prepared from the remaining in vitro polyadenylated RNAs (∼4 ng) using the PCR-cDNA barcoding sequencing kit from Oxford Nanopore (ONT, catalog #: SQK-PCB111.24) as per the manufacturer’s instructions. Sequencing was performed using R9.4.1 Flongle cells on a MinION Mk1B device and sequenced for 24 h. Basecalling was performed using Guppy Basecaller (version 6.1.1+1f6bfa7f8). Reads were then mapped to the human mitochondrial genome (see Fig S3) using Minimap 2 (version 2.17-r941). Reads were visualized using IGV (version 2.12.3), and figures were prepared using Inkscape (version 1.1.2). Data are available as FASTQ files and aligned bam files.

### dsRNA sequencing and analysis in cancer cell lines

A cell lysate was treated with DNase I to degrade DNA followed by a 1-h incubation at 37°C with Nuclease S1 RNase to remove single-stranded RNA. RNA was purified and incubated with 50% DMSO vol/vol for 1.5 h at 65°C to minimize the secondary structure, then purified with RNeasy MinElute Cleanup Kit (QIAGEN). Strand-specific NGS libraries were constructed from the remaining dsRNA using the KAPA RNA HyperPrep kit (Roche) and sequenced on the Illumina NextSeq using 2 × 100 bp paired-end reads.

The dsRNA-sequencing data were processed using nf-core/rnaseq pipeline, version 3.8 (Ewels et al, 2020), with the following options: reference genome, hg19; adapter and quality trimming, Trim Galore; alignment and quantification route, STAR>Salmon; sort and index alignments, SAMtools; duplicate read marking, Picard MarkDuplicates.

The strandCheckR package (To & Pederson, 2021) was used to quantify the proportion of reads originating from double-stranded RNA. First, a 100-bp window that slides over a bam file (window step = 20 bp) was used to determine the proportion of the genome with reads mapping to both the “+” and “−” strands for each window (i.e., symmetrically aligned reads). Windows that primarily include reads derived from single-stranded DNA (i.e., asymmetrically aligned reads) were eliminated. For each sliding window, the proportion of ± stranded reads was computed. Windows with more than 70% of reads from a single strand (e.g., 25% positive, 75% negative) were classified as having single-strand coverage. The remaining window was deemed coverage for double-strand RNA. Coverage of double-strand RNA was adjusted by total coverage per chromosome. Visualization of the coverage per mitochondrial region was performed using Tableau.

### Quantitative PCR analysis for gene expression

Cells were lysed by drawing through a sterile 21-gauge needle and syringe to homogenize the cell. The RNA from the total lysate was isolated using the QIAGEN RNeasy spin column kit according to the manufacturer’s instructions. RNA was reverse-transcribed into cDNA using Bio-Rad iScript cDNA Synthesis Kit (Bio-Rad) in accordance with the manufacturer’s protocols. Reverse transcriptase quantitative PCR was performed with the 2x SYBR Green qRT-PCR kit (Kapa Biosystems) and analyzed with the Roche LightCycler 480 II system (Roche). qRT-PCR primers are listed in Table S3. Samples from three biological replicates were plated for three technical replicates, and the average raw cycle threshold value across the three replicates was calculated.

Table S3. Primers for qRT-PCR analysis used in this study.

## Supplementary Material

Reviewer comments
