# Supplementary material for: Trafficking of mitochondrial double-stranded RNA from mitochondria to the cytosol
Source: Life Sci Alliance. 2024 Jul 2;7(9):e202302396. doi: 10.26508/lsa.202302396 (PMC11220484; doi:10.26508/lsa.202302396)
Supplement: Supplementary file 1 [file LSA-2023-02396_TableS1.docx]

**Table S1. siRNA information used in this study**

| **Target Gene** | **Target Sequence** | **siRNA Type** | **Vendor** |
| --- | --- | --- | --- |
| Silencer Select siRNA #1 (Neg Control) | N/A | silencer select siRNA | Invitrogen |
| PNPT1 | HSS131758, HSS131759 | stealth RNAi siRNA | Invitrogen |
| mouse PNPT1 | s232318 | silencer select siRNA | Ambion |
| SUV39H2 | s36184 | silencer select siRNA | Invitrogen |
| GHITM | s25763 | silencer select siRNA | Ambion |
| VDAC1 | s14768 | silencer select siRNA | Ambion |
| VDAC2 | s14771 | silencer select siRNA | Ambion |
| PHB1 | s10424 | silencer select siRNA | Ambion |
| PHB2 | s22344 | silencer select siRNA | Ambion |
| CLIC5 | 135160 | silencer siRNA | Ambion |
