# Supplementary material for: Trafficking of mitochondrial double-stranded RNA from mitochondria to the cytosol
Source: Life Sci Alliance. 2024 Jul 2;7(9):e202302396. doi: 10.26508/lsa.202302396 (PMC11220484; doi:10.26508/lsa.202302396)
Supplement: Supplementary file 2 [file LSA-2023-02396_TableS2.docx]

**Table S2. Antibodies used in this study**

| **Antibody Specificity** | **Antibody Species** | **Antibody Vendor** | **Catalog Number** |
| --- | --- | --- | --- |
| TOMM40 | Rabbit | Proteintech | 18409-1-AP |
| TOMM20 | Rabbit | Proteintech | 11802-1-AP |
| TIA1 | Rabbit | Proteintech | 12133-2-AP |
| J2 | Mouse | Exalpha | 10010500 |
| SUV3 | Rabbit | Bethyl Laboratories | A303-056A |
| MDA5 | Rabbit | Thermo Fisher | PA5-18164 |
| PNPase | Rabbit | Pacific Immunology | 3370T, (Chen HW et al, 2006, Rainey RN et al, 2006) |
| Rabbit Secondary Ab | Rabbit | Thermo Fisher | 0031461 |
| Mouse Secondary Ab | Mouse | Thermo Fisher | 0031430 |
| 488 AlexaFluor Rabbit Secondary Ab | Rabbit | Abcam | ab150077 |
| 488 AlexaFluor Mouse Secondary Ab | Mouse | Invitrogen | A11029 |
| 568 AlexaFluor Rabbit Secondary Ab | Rabbit | Invitrogen | A11036 |
| 568 AlexaFluor Mouse Secondary Ab | Mouse | Abcam | ab175473 |
