# Supplementary material for: Trafficking of mitochondrial double-stranded RNA from mitochondria to the cytosol
Source: Life Sci Alliance. 2024 Jul 2;7(9):e202302396. doi: 10.26508/lsa.202302396 (PMC11220484; doi:10.26508/lsa.202302396)
Supplement: Supplementary file 3 [file LSA-2023-02396_TableS3.docx]

**Table S3. Primers for rRT-PCR Analysis used in this study.**

| **Primer** | **Forward Sequence** | **Reverse Sequence** | **Vendor** |
| --- | --- | --- | --- |
| IFNB1 | ATGACCAACAAGTGTCTCCTCC | GCTCATGGAAAGAGCTCTAGTG | IDT |
| ISG15 | ACTCATCTTTGCCAGTACAGG | CAGCTCTGACACACCGACATG | IDT |
| IFI44 | GTAACGCATCAGGCTTTGGT | CCGCCTTCTTTCTCACTCAG | IDT |
| MDA5 | TCGAATGGGTATTCCACAGACG | GGTGGCGACTGTCCTCTGAA | IDT |
